# A leptin-based Bayesian inference of a pro-satiety state reflects a basal circadian rhythm in women with obesity

**DOI:** 10.3389/fendo.2025.1638568

**Published:** 2025-09-16

**Authors:** Qing Xiang, Saman Khazaei, Rose T. Faghih

**Affiliations:** Department of Biomedical Engineering, Tandon School of Engineering, New York University, New York, NY, United States

**Keywords:** satiety, leptin, obesity, marked point process, Bayesian filtering, circadian rhythm

## Abstract

**Introduction:**

Leptin, primarily secreted by adipose tissue, is a critical hormone involved in regulating energy balance and food intake by inducing satiety. Although several hormones influence satiety, leptin plays a dominant role in long-term satiety regulation.

**Methods:**

We apply a state-space estimation framework using Bayesian filtering to infer continuous, long-term pro-satiety states from plasma leptin concentrations collected from premenopausal women with obesity. Our approach adopts methodologies previously applied to biosignals such as skin conductance and cortisol data to estimate latent states, leveraging the features in the leptin secretory pulses and plasma leptin levels. Additionally, we investigate the potential influence of meals, sleep, and bromocriptine treatment on the pro-satiety states. We introduce the High Satiety Index (HSI), a direct, long-term satiety measure based on leptin secretion dynamics, minimizing biases inherent in conventional assessment methods.

**Results:**

Comparisons of the estimated state in different time windows show that the pro-satiety state inferred by leptin secretion is significantly higher during sleep, aligning with a circadian rhythm. The estimated state does not show a significant variation in response to meal intake or bromocriptine treatment.

**Discussion:**

The leptin-based estimator reflects basal variations of satiety in women with obesity. This framework shows the feasibility of applying Bayesian filtering to track satiety and can be further developed to perform a multimodal estimation.

## 1 Introduction

Leptin is a hormone secreted primarily in the adipose tissues (fat cells) and is associated with body weight (1, 2). It regulates body energy and food intake by inducing satiety (1, 3–6). Although other hormones such as gut hormones also affect satiety, leptin is a major factor that affects long-term satiety (7, 8). Leptin provides information on the energy storage in the human body to the brain. As fat accumulates, more leptin is released and acts in the hypothalamus, leading to high satiety and behaviors that will reduce energy intake (9). Previous studies have conducted and discussed several methods of satiety measurement, including bipolar and unipolar scales (10) and appetite ratings using the visual analogue scale (VAS) (11, 12). However, these measurements are subjective and may require consideration of various conditions under which the measurements are taken (10). The measurement of satiety is often under the guideline of the satiety cascade, first introduced by Blundell et al. in (13). The cascade describes short-term and long-term aspects that affect satiety. It also distinguishes short-term and long-term satiety by naming the former “satiation” and the latter “satiety” (13). The former refers to the feeling that stops eating, meaning that it is mainly affected by sensory factors such as gastric distension (7). Satiety, or more precisely long-term satiety, on the other hand, determines the beginning of meals and is often considered the opposite of hunger (14). Long-term satiety is often measured between meals, but the measurement is usually complicated by many internal and external factors (7).

Other hormones are also involved in the satiety cascade, such as ghrelin, a hunger-inducing hormone (13, 14). Ghrelin is secreted from the stomach and has a short-term effect of increasing appetite (15). It does not affect leptin but acts as a counterpart to leptin and can be seen as an anti-satiety feature (16), as they both act in the hypothalamus and affect downstream neural signaling that leads to food related behaviors (8). Leptin’s primary role is to regulate energy storage in the body, but its regulation is asymmetric, as it is more effective in replenishing fat storage when it is low than resisting obesity (8). Giving leptin’s characteristics, our goal is to decode a continuous pro-satiety state from plasma leptin concentration collected from a cohort of women with obesity, as an effort to measure the long-term basal satiety in an objective way.

Many latent state estimation schemes have been developed based on state-space models. Information extracted from biosignals and behavior measurements has been used as observations from which the state is estimated (17). These state-space methods have been applied in various fields including behavioral learning (18–21), movement decoding (22, 23), latent state underlying neural spiking estimation (24, 25), and sleep onset process tracking (26). The estimators developed in these studies often utilized Bayesian filtering for processing binary observations. Moreover, Wickramasuriya et al. (27) introduced a marked point process (MPP) plus continuous observation estimator that takes the binary sequence of impulse occurrences underlying sweat secretion as one of the observations to estimate the hidden sympathetic arousal state. In this research, we implement a similar state-space approach, leveraging information extracted from plasma leptin measurements and utilizing Bayesian filtering.

**Figure 1:**
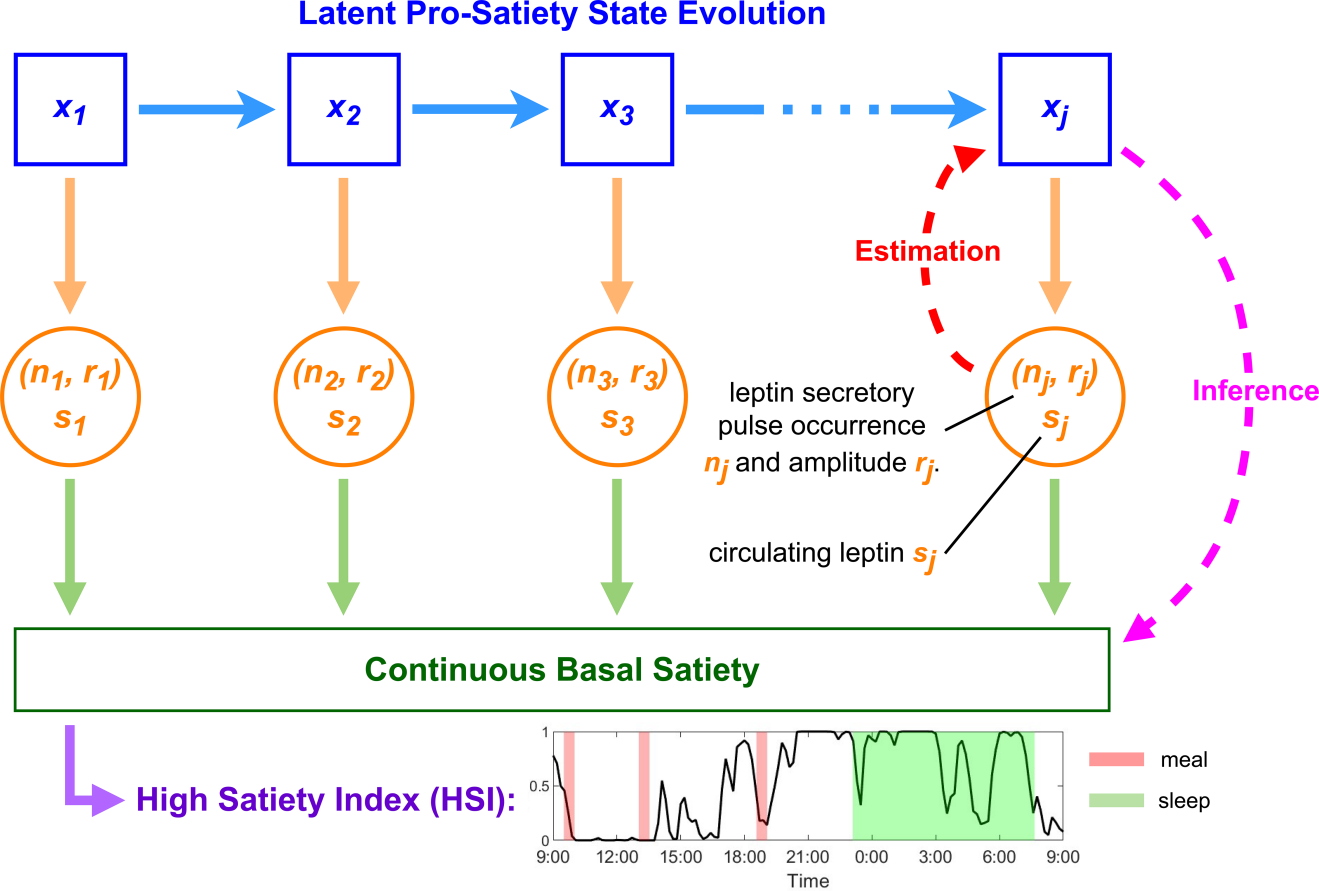
Pro-satiety state evolution and basal satiety inference scheme. *x_j_* is the leptin-related hidden pro-satiety state variable at time instant *j* to be estimated. *n_j_*, *r_j_*, and *s_j_* are observations and denote leptin pulse occurrence, leptin pulse amplitude, and plasma leptin concentration, respectively.

Similar to skin conductance signals that arise from sweat secretion by sweat glands near the measurement site, blood leptin comes from fat cells (1) and can be modeled by a two-compartment state-space model (28). In addition, leptin pulse amplitudes and inter-pulse intervals have similar distributions to sweat secretory pulses (29, 30). The estimator applies Bayesian causal inference which is well-suited for decoding the latent arousal state from skin conductance and latent energy state from cortisol given the causal links (27). But this estimator cannot directly decode long-term satiety from leptin data. Unlike the relationship between the arousal state and sweat secretion, where the hidden state causes the secretion, satiety is an effect of leptin (6), which means that the direction of causality is the opposite. However, we assume a latent pro-satiety state that controls leptin secretion, and this pro-satiety state can be an indicator of a person’s overall satiety. Since the state estimation scheme assumes a causal link between the hidden state and leptin secretion, and leptin controls long-term satiety (5, 6), the estimated state based on leptin may display the dynamics of a person’s basal satiety over time. **Figure 1** illustrates our proposed satiety inference scheme.

After state estimation, to investigate the latent state’s potential association with food intake, we compare the estimated state before, during, and after each meal. We also compare wake and sleep periods since it has been reported that leptin follows a circadian rhythm (4, 31). The original study of the dataset used in this study was designed partly to test the effect of bromocriptine on obesity (32). With this satiety inference framework, we also examine the effect of bromocriptine treatment on the estimated pro-satiety state during these mentioned windows, providing a new perspective in testing the drug’s effect. We define a High Satiety Index (HSI) that provides a judgment of the participant’s basal satiety status based on the estimated state. With this objective method of satiety evaluation, continuous tracking and automated control systems can be implemented in portable devices to monitor and influence a person’s satiety. It is also a step toward a multimodal method of satiety inference and a monitor for dynamic latent health status in the human body.

## 2 Materials and methods

### 2.1 Dataset

We use leptin data collected in the previous clinical studies by Kok et al. (32–34). The original clinical study was approved by the Medical Ethics Committee of the Leiden University Medical Center and conducted based on the Helsinki Declaration (32). The written acknowledgment of informed consent for participation was obtained from all participants. The participants were 18 healthy premenopausal women with obesity (BMI 30.1–40.5*kg/m* ^2^, age 22–51 years with mean = 37.5 ± 1.7) who were not under the influence of any medication or drugs. Participants with acute or chronic disease, depression, head trauma, habits of smoking or alcohol consumption, recent transmeridian flights, nightshift work, weight change, blood donation, or participation in another clinical trial were excluded. The data were gathered during the early follicular phase of their menstrual cycles confirmed by plasma estradiol and progesterone levels, to minimize hormonal variability between individuals and across measurements. All, and all participants were served with the same meal and had a strict meal and sleep schedule. The meals consist of liquids (16% proteins, 49% carbohydrates, and 35% fat) for a total of 2100 kilocalories per day. The meals periods are from 09:30 to 10:00, 13:00 to 13:30, and 18:30 to 19:00. The sleep period is from 23:00 to 07:30 on the next day. The trial is 24 hours long, from 09:00 to 09:00 on the next day, following 7 days of placebo treatment. Plasma leptin concentration levels were measured from blood samples using radioimmunoassay, which has a detection limit of 0.5 ng/L, at 10-minute intervals throughout this period. The same protocol was repeated four weeks later with the same participants who received a 2.5 mg dose of bromocriptine twice per day for 7 days this time.

In their study, Reddy et al. (28) extracted the underlying secretory pulses as an MPP through a deconvolution method based on a state-space model. They also reconstructed the leptin level using the MPP. In this study, we utilize both the extracted MPP and the reconstructed leptin level as inputs to the estimator.

### 2.2 Leptin-related pro-satiety state estimation

To estimate the hidden pro-satiety states that give rise to leptin secretory events, we employ an MPP estimator with continuous observation that involves the inter-pulse intervals, pulse amplitudes, and the plasma leptin level altogether. Unlike a binary estimator that treats the secretory pulse sequence as a point process where pulse amplitudes are ignored (35) or an MPP estimator that estimates the hidden state based on the MPP only (17), this approach assumes linear relationships between both the hidden state and the MPP and between the state and the plasma leptin level. In their previous study, Wickramasuriya et al. applied such an estimator to estimate arousal state based on skin conductance and energy state based on blood cortisol level (27). The leptin pulses studied in this paper were recovered based on the same state-space model structure as applied in the estimation of skin conductance and cortisol pulses (27, 28). Since there are very few pulse events (29 to 45) compared to the total length of 1441 time instants where a pulse can occur, a substantial portion of the MPP is 0 which trivializes the nonzero amplitudes (28). To solve this, we chose larger time bin sizes based on the minimum time interval between pulse events for each MPP and resampled the data. We use the same state dynamics and algorithm to decode leptin secretion. The state dynamics is:


(1) $$x_{j}=x_{i-1}+\epsilon_{j},$$

where *x_j_* is the state at time instant *j* and $\epsilon_{j}∼N(0,\sigma_{\epsilon }^{2})$ is the process noise. This definition assumes a random walk process that controls the state’s evolution over time, which was chosen to model neural states in several previous works as well as the skin conductance study (18–20, 24, 26). We denote the occurrence of a pulse in the MPP as *n_j_* ∈ (0,1). *n_j_* is a Bernoulli-distributed random variable with mass density $p{\left(n_{j}=1\right)}^{n_{j}}(1-p{\left(n_{j}=1\right)}^{1-n_{j}})$ . Let $p_{j}=p(n_{j}=1)$ , then *p_j_* is related to the state variable *x_j_* via a logit transformation according to the theory of generalized linear models (36):


(2) $$\log\;(\frac{p_{j}}{1-p_{j}})=\beta_{0}+\beta_{1}x_{j},$$

(3) $$p_{j}=\frac{1}{1+e^{\left(-\beta_{0}+\beta_{1}x_{j}\right)}},$$

where *β*_0_ and *β*_1_ are coefficients to be determined.

We further define the linear relationship between the leptin secretory pulse amplitudes *r_j_* and the state variable *x_j_* as:


(4) $$r_{j}=\gamma_{0}+\gamma_{1}x_{j}+\nu_{j},$$

where *γ*_0_ and *γ*_1_ are coefficients to be determined, and $\nu_{j}∼N(0,\sigma_{\nu }^{2})$ is the measurement noise (27).

Based on the binary variable *n_j_* and the continuous variable *r_j_* the joint probability density function for an observation at a time instant given the hidden state is,


(5) $$p(n_{j}\cap r_{j}|x_{j})=\{\begin{array}{ll} 1-p_{j}, & \text{if}\;n=0,\\ \frac{p_{j}}{\sqrt{2\pi \sigma_{\nu }^{2}}}e^{\frac{-{\left(r_{j}-\gamma_{0}-\gamma_{1}x_{j}\right)}^{2}}{2\sigma_{\nu }^{2}}}, & \text{if}\;n=1. \end{array}$$

This is based on the assumption that the distribution of the amplitudes of the leptin secretory pulses can be modeled by a Gaussian model, which is proven to be effective in our previous study (30). The Gaussian distribution is also assumed to be a proper model for the distribution of the skin conductance pulses (35). When no pulse is observed, *r_j_* is not involved.

In addition to *r_j_*, we relate the reconstructed plasma leptin level *s_j_* to the state variable *x_j_* via:


(6) $$s_{j}=\delta_{0}+\delta_{1}x_{j}+\omega_{j},$$

where *δ*_0_ and *δ*_1_ are coefficients to be estimated, and $\omega_{j}∼N(0,\sigma_{\omega }^{2})$ is the measurement or modeling noise (27). The original continuous-valued leptin level from plasma measurements has a lower resolution (10-minute time bin size) than the extracted underlying pulses (1-minute time bin size). To address this problem, we reconstruct the continuous-valued leptin level using the extracted pulses. The reconstructed results according to the state-space model of leptin secretion defined in the previous chapter closely resemble the original measurement (28). Now the state *x_j_* gives rise to both the MPP observation and the plasma leptin level observation with 1-minute time bin size. Although *x_j_* is linearly related to both the MPP and the reconstructed leptin level, the overall model is nonlinear due to *x_j_*’s relationship with *p_j_* (27).

To estimate the hidden state *x_j_* and $\sigma_{\epsilon }^{2}$ , we apply an expectation-maximization (EM) algorithm. The EM algorithm consists of two steps: E-step, where we predict the current state and the process noise limit based on the initial or previous state prediction; and M-step, where we estimate the parameters that maximize the likelihood of the predicted state variable. Based on Equations 1–6, the posterior density function for the E-step is (27):


(7) $$p(x_{j}|y_{1:j})=\frac{p(x_{j}|y_{1:j-1})p(n_{j}\cap r_{j}|x_{j})p(s_{j}|x_{j})}{p(y_{j}|y_{1:j-1})},$$

where *y_j_* denotes the collective observations of the MPP and the reconstructed leptin level.

#### 2.2.1 E-Step

Based on Equation 7, we implement Bayesian filtering and utilize both a forward filter and a backward smoother in the E-step, as applied in the skin conductance study (17, 27, 35). The forward filter estimates $x_{j|j}$ using all previous observations $n_{1:j}$ , and the backward smoother estimates $x_{j|J}$ using all observations $n_{1:J}$ (35). The following shows one iteration of the E-step which involves a Gaussian approximation (18, 35).

*Predict:*


(8) $$x_{j|j-1}=x_{j-1|j-1},$$

(9) $$\sigma_{j|j-1}^{2}=\sigma_{j-1|j-1}^{2}+\sigma_{\epsilon }^{2},$$

*Update:*

if *n_j_* = 0


(10) $$C_{j}=\frac{\sigma_{j|j-1}^{2}}{\delta_{1}^{2}\sigma_{j|j-1}^{2}+\sigma_{\omega }^{2}},$$

(11) $$\begin{array}{c} x_{j|j}=x_{j|j-1}+C_{j}[\sigma_{\omega }^{2}\beta_{1}\left(n_{j}-p_{j|j}\right)\\ +\delta_{1}\left(s_{j}-\delta_{0}-\delta_{1}x_{j|j-1}\right)], \end{array}$$

(12) $$\sigma_{j|j}^{2}={\left[\frac{1}{\sigma_{j|j-1}^{2}}+\beta_{1}^{2}p_{j|j}\left(1-p_{j|j}\right)+\frac{\delta_{1}^{2}}{\sigma_{\omega }^{2}}\right]}^{-1},$$

if *n_j_* = 1


(13) $$C_{j}=\frac{\sigma_{j|j-1}^{2}}{\sigma_{\nu }^{2}\sigma_{\omega }^{2}+\sigma_{j|j-1}^{2}(\gamma_{1}^{2}\sigma_{\omega }^{2}+\delta_{1}^{2}\sigma_{\nu }^{2})},$$

(14) $$x_{j|j}=x_{j|j-1}+C_{j}[\sigma_{\nu }^{2}\sigma_{\omega }^{2}\beta_{1}(n_{j}-p_{j|j})+\gamma_{1}\sigma_{\omega }^{2}(r_{j}-\gamma_{0}-\gamma_{1}x_{j|j-1})+\delta_{1}\sigma_{\nu }^{2}(s_{j}-\delta_{0}-\delta_{1}x_{j|j-1})],$$

(15) $$\sigma_{j|j}^{2}={\left[\frac{1}{\sigma_{j|j-1}^{2}}+\beta_{1}^{2}p_{j|j}(1-p_{j|j})+\frac{\gamma_{1}^{2}}{\sigma_{\nu }^{2}}+\frac{\delta_{1}^{2}}{\sigma_{\omega }^{2}}\right]}^{-1}.$$

Note that since *p_j_*_|_ *_j_* is calculated using *x_j_*_|_ *_j_* (11), and (14) has *x_j_*_|_ *_j_* on both sides of the equation which is solved numerically by the Newton-Raphson method. Besides, the algorithm switches between two ways of updating based on *n_j_*’s value. This switching is to account for the different cases in (5) for the MPP.

After predicting the state variable and the variance at each time bin, we improve the predictions by applying the backward smoother (17, 27, 35, 37):


(16) $$A_{j}=\frac{\sigma_{j|j}^{2}}{\sigma_{j+1|j}^{2}},$$

(17) $$x_{j|J}=x_{j|j}+A_{j}(x_{j+1|J}-x_{j+1|j}),$$

(18) $$\sigma_{j|J}^{2}=\sigma_{j|j}^{2}+A_{j}^{2}(\sigma_{j+1|J}^{2}-\sigma_{j+1|j}^{2}).$$

#### 2.2.2 M-Step

We derive $\gamma_{0},\gamma_{1},\delta_{0},\delta_{1},\sigma_{\nu }^{2},\sigma_{\omega }^{2},\sigma_{\epsilon }^{2}$ by maximizing the expected log-likelihood *L* defined as


(19) $$\begin{array}{c} L=\sum_{j=1}^{J}n_{j}(\beta_{0}+\beta_{1}x_{j})-\log\;(1+e^{\beta_{0}+\beta_{1}x_{j}})\\ -\frac{\left|\tilde{J}\right|}{2}\log\;(2\pi \sigma_{v}^{2})-\sum_{j\in \tilde{J}}\frac{{\left(r_{j}-\gamma_{0}-\gamma_{1}x_{j}\right)}^{2}}{2\sigma_{v}^{2}}\\ -\frac{J}{2}\log\;(2\pi \sigma_{\omega }^{2})-\sum_{j=1}^{J}\frac{{\left(s_{j}-\delta_{0}-\delta_{1}x_{j}\right)}^{2}}{2\sigma_{\omega }^{2}}\\ -\frac{J}{2}\log\;(2\pi \sigma_{\epsilon }^{2})-\sum_{j=1}^{J}\frac{{\left(x_{j}-x_{j-1}\right)}^{2}}{2\sigma_{\epsilon }^{2}}, \end{array}$$

where $\tilde{J}$ denotes the sequence of locations where pulses occur. To estimate *β*_0_ and *β*_1_, we apply one of the two approaches mentioned in (27). From Equation 2, we have


(20) $$\beta_{0}=\log\;(\frac{p_{0}}{1-p_{0}}),$$

if *x_j_* = 0 at time *j*. We set *β*_1_ = 1 and assume that *x*_0_ ≈ 0, and the baseline probability *p_b_* approximates *p*_0_. *p_b_* is taken to be the number of pulses in the MPP divided by the length of the MPP:


(21) $$p_{b}=\frac{\sum_{j=1}^{J}n_{j}}{J}$$

The algorithm based on Equations 8–21 iterates between the two steps until convergence, which we consider is achieved when the mean distance between obtained variables in consecutive iterations is smaller than 10^−8^ (17, 18, 27, 35).

### 2.3 High satiety index

Satiety index has been given to food, suggesting how much satiety different types of food can induce in a person (38). Inspired by the High Arousal Index defined in (35), we define the High Satiety Index (HSI) based on the estimated pro-satiety state in humans as mentioned before. The HSI is expressed as the probability of the estimated state exceeding a certain threshold. It indicates the certainty of the participant having high satiety based on observation, similar to the ideal observer certainty defined in (18). We set the threshold to be the median of the state value so that it may illustrate a potentially significant change from wakefulness to sleep period during a full day. Since the estimated state reflects long-term satiety, the HSI indicates the baseline change of the participant’s satiety. When the HSI is near 1, it suggests that the participant is very likely satiated at that time and has no appetite, whereas when it is near 0, the participant potentially wants to start eating. It is worth noting that the HSI might not reflect the direct feeling of hunger or fullness which are more related to short-term satiety or satiation (14).

## 3 Results

We applied an MPP with a continuous observation estimator to uncover the leptin-related hidden state of the 18 participants. The estimator assumed causal links both between the hidden state and the leptin secretory pulses and between the hidden state and the reconstructed plasma leptin levels. Through the EM algorithm, we obtained the estimated pro-satiety state over 24 hours for the pre-treatment and post-treatment samples of each participant. For comparison, we also modified the estimator to apply two other versions. One only considers the MPP and the other only considers the continuous observation. The solely MPP-based estimator was implemented in (35), where the authors linked the pulsatile sweat secretion modeled as an MPP to the sympathetic arousal state. The estimator design is the similar to the estimator described above where a logit transformation is applied to linearly link the pulse probability to the state variable. Similarly the other version that is solely based on the continuous observation considers the relationship in 6 alone, thus modifying the update equations and cost function in the EM algorithm. The overall structure remains the same in both versions and their results are compared to the main estimator, which is based on both MPP and continuous observation.

**Figure 2:**
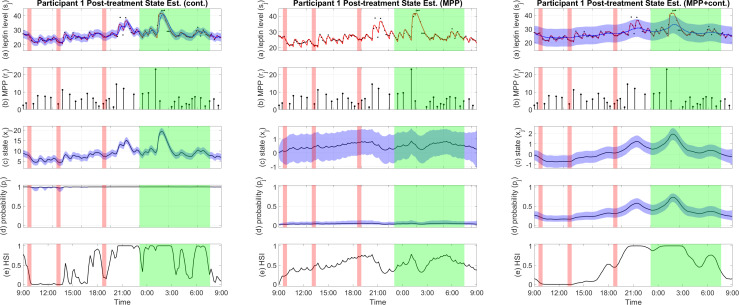
Examples of pro-satiety state estimation results by different estimators. Figures from left to right are estimator based on continuous observation, estimator based on MPP, and estimator based on MPP plus continuous observation, respectively. **(A)** Plasma leptin measurements (black), reconstructed plasma leptin concentration level from deconvolution result (red), and reconstructed plasma leptin concentration level from state estimate with a 95% confidence interval (blue). **(B)** Leptin secretory pulse events. **(C)** Estimated state with a 95% confidence interval. **(D)** Probability of leptin pulse occurrence with a 95% confidence interval. **(E)** The High Satiety Index (HSI). Meal periods are highlighted in red, and sleep period is highlighted in green. Est, estimation; Cont, continuous observation; MPP, marked point process.

The results show that the estimated state based on MPP alone does not vary much over time compared to the other two versions while having relatively wide confidence intervals. An example is shown in **Figure 2**. From the solely MPP-based estimator, the estimated probability of pulse occurrence also has little oscillations through our the day, while the HSI stays close to the middle for the majority of the time. On the other hand, the estimator solely based on the continuous observation overfits the data. At the same time, the MPP plus continuous observation estimator balances the variances of the MPP and the leptin levels to output a meaningful result that reflects changes in both inputs and avoids overfitting.

Since no pulse sequence is involved in the continuous observation estimator, pulse occurrence probability is not meaningful; likewise, due to the lack of a relationship between the leptin levels and the hidden state, we cannot reconstruct leptin levels from the state estimates in the MPP estimator. A comparison of the results produced by these three versions of estimators for an example participant is shown in **Figure 2**. Results for all participants are available in the **Supplementary Materials**.

**Figure 3:**
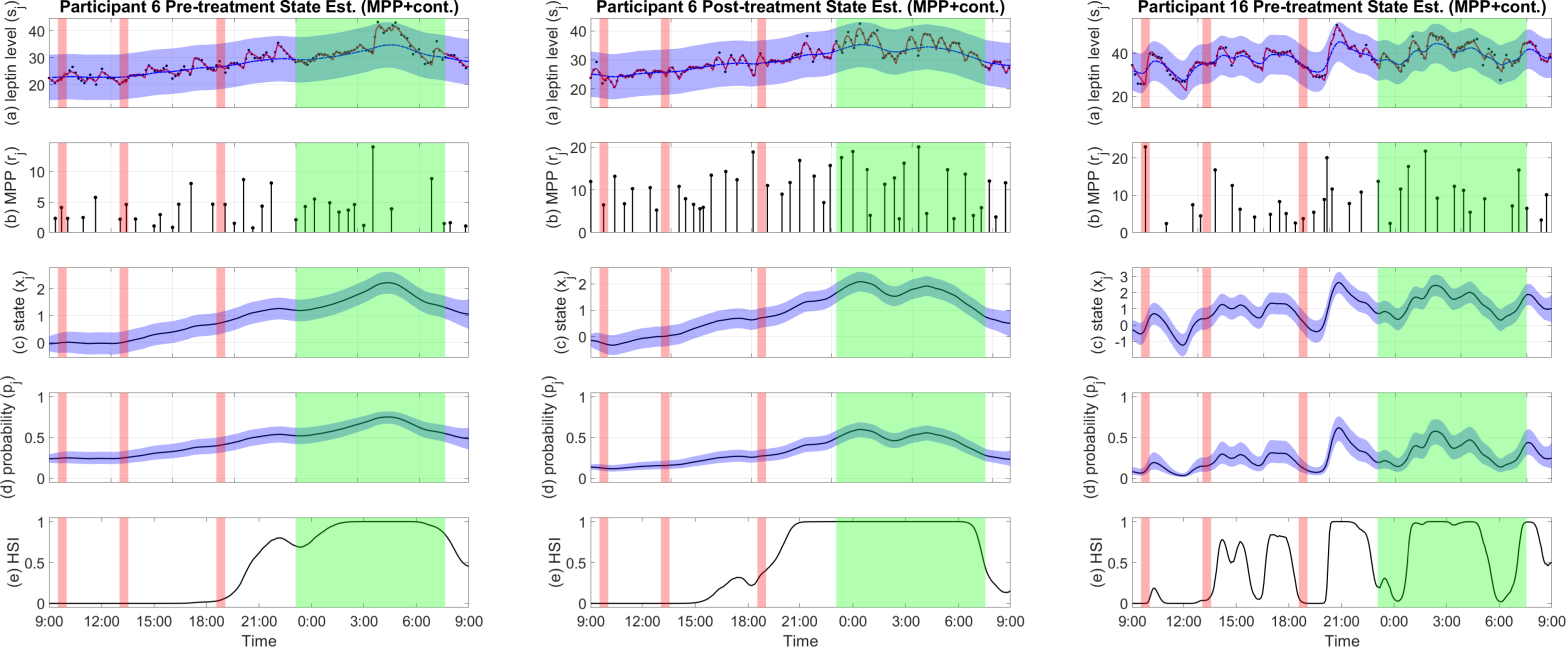
Examples of pro-satiety state estimation results of the MPP plus continuous observation estimator. **(A)** Plasma leptin measurements (black), reconstructed plasma leptin concentration level from deconvolution result (red), and reconstructed plasma leptin concentration level from state estimate with a 95% confidence interval (blue). **(B)** Leptin secretory pulse events. **(C)** Estimated state with a 95% confidence interval. **(D)** Probability of leptin pulse occurrence with a 95% confidence interval. **(E)** The High Satiety Index (HSI). Meal periods are highlighted in red, and sleep period is highlighted in green. Est, estimation; Cont, continuous observation; MPP, marked point process.

In the results of the MPP plus continuous estimator, we saw a dominant pattern of increased plasma leptin level during sleep in all samples, regardless of the bromocriptine treatment (28) as shown by Participant 6’s state estimation result in **Figure 3**. The estimated state shows a general trend that consists of an increase during the day and a rapid decrease before waking, with a peak around mid-sleep and a nadir in the morning.

This is also reflected by the HSI in the majority of cases. Example estimation results are shown in **Figure 3**. Estimation results of other participants can be found in the **Supplementary Materials**.

**Figure 4:**
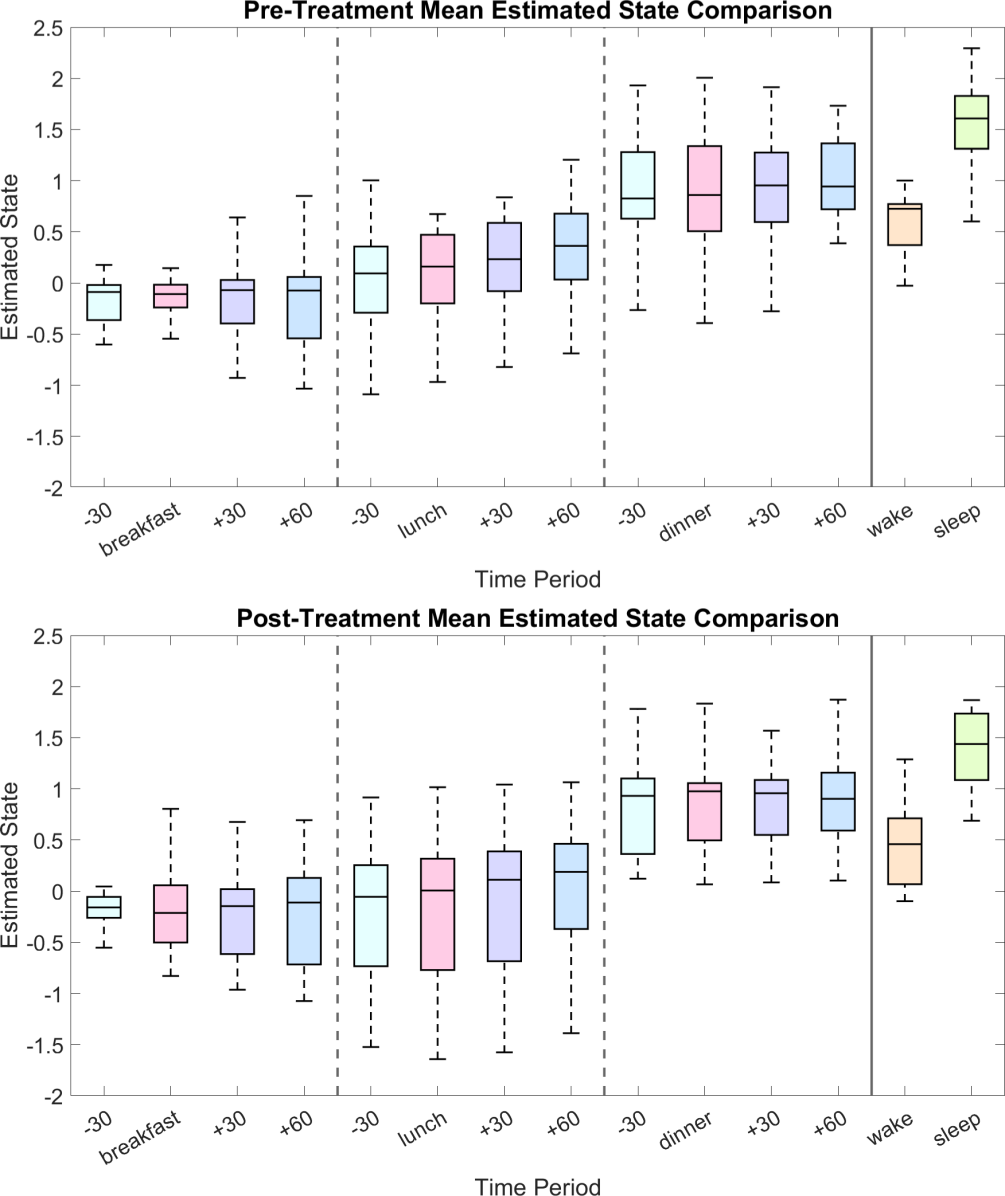
Mean pro-satiety state values across distinct time windows relative to meals. Each meal window (“breakfast,” “lunch,” and “dinner”) represents the 30-minute period during which participants had their first, second, and last meals of the day, respectively. Other 30-minute windows include “-30” (from 29 minutes before to the start of each meal), “+30” (1 to 30 minutes following each meal), and “+60” (31 to 60 minutes following each meal). Box plots covering the wake (07:31–23:00) and sleep (23:01–07:30) periods are also shown.

To evaluate potential meal influence on the pro-satiety state, we calculated the mean state value during different meal-related time periods. **Figure 4** shows the distribution box plots of the mean state values of all participants. Specifically, in these time windows, a participant might be preparing to eat, eating, or digesting food. Comparing the estimated state during these time windows may inform pro-satiety state changes due to having meals. According to the trial design that includes a strict schedule for meals, we focus on 4 time windows for each meal:

1. 29 minutes to 0 minutes before the start of the meal period (-30 window),
2. the 30-minute meal period (meal window),
3. 1 minute to 30 minutes after the end of the meal period (+30 window), and
4. 31 minutes to 60 minutes after the end of the meal period (+60 window).

We expect that the state values will not show significant change during each meal-related window than in all previous windows, since our estimator is solely leptin-based which is not sensitive to food intake in a short period (7). To test if there is an increase or decrease and if the increase or decrease is consistent during and one hour after meals, we made six comparisons with the median state value for each meal-related time window. Specifically, we perform a two-tailed Wilcoxon signed rank test (39) on each pair of data to test if they are different with statistical significance.

According to **Table 1** and **Figure 4**, there is a consistent increase for at least one hour since the beginning of lunch. The distribution of state values during the lunch related windows seem to spread out more across all participants after bromocriptine treatment, but the general increase remained statistically significant. Besides, both pre-treatment and post-treatment data show a much higher mean state value during sleep than during wakefulness (*p* = 0.0002 and *p* = 0.0033). Wilcoxon signed rank tests comparing pre-treatment and post-treatment data over the same time periods show no statistical significant results.

**Table 1:** *P*-values of two-tailed Wilcoxon signed rank test on estimated state from each two meal-related windows.

Meals	*meal* vs.-30	+30 vs.-30	+60 vs.-30	+30 vs.*meal*	+60 vs.*meal*	+60 vs. +30
Breakfast pre-treatment	0.3491	0.3061	0.3720	0.6165	0.5566	0.3271
Breakfast post-treatment	0.1570	0.1701	0.1989	0.1701	0.2668	0.5566
Lunch pre-treatment	**0.0096**	**0.0123**	**0.0043**	**0.0123**	**0.0005**	**0.0002**
Lunch post-treatment	**0.0249**	**0.0004**	**0.0002**	**0.0002**	**0.0002**	**0.0002**
Dinner pre-treatment	**0.0347**	0.1119	0.1570	0.3958	0.3491	0.2311
Dinner post-treatment	0.1989	0.2145	0.0854	0.2145	**0.0347**	**0.0429**

*meal*: meal window; −30: from 29 minutes before to the start of a meal; +30: 1 to 30 minutes following a meal; +60: 31 to 60 minutes following a meal. *p*-values less than 0.05 are in bold (indicating statistical significance).

## 4 Discussion

Using the MPP plus continuous observation estimator, we obtain the hidden pro-satiety state that leads to the observation of both the underlying leptin secretory pulses and the plasma leptin levels. We also applied an MPP-based estimator without linking the state to the reconstructed leptin levels. As demonstrated in **Figure 2**, this estimator does not produce state values that have large variations over time compared to the results from the other two estimators. This is likely due to the fact that leptin pulses are more spread out throughout the day and do not have a burst during any short time window. To strengthen the influence of the leptin secretory events, we downsampled the pulse sequences by dividing them into segments and using a single pulse to represent all pulses in each segment with an aggregated pulse amplitude. This helps concentrate the influence of each pulse. However, since almost no inter-pulse intervals are very small (30), the estimated state is still relatively flat after downsampling the pulse sequence. For estimating the pro-satiety state, adding the continuous leptin concentration as a reference would make the estimated state more informative, as it would show additional features which might directly reflect the impact of leptin in the hypothalamus, leading to downstream satiety related neural activities (8).

A simple state estimator based on continuous observation alone can extract important trends from the data. However, this estimator leaves out the influence of the timings and amplitudes of leptin secretory pulses, and thus cannot produce meaningful probabilities for the pulse occurrences. Algorithmically, since the estimator also updates and minimizes the variances of the input, it quickly overfits and presents a convergence issue. The formulation of the estimator involves a forward filter and a backward filter. Without the MPP, the forward filter resembles a Kalman filter that reduces noise based on the variance of the input (40, 41). However, since the estimator also minimizes the input variance, it overfits the input as the variance approaches 0. On the other hand, the output of the MPP plus continuous observation estimator outputs a state that reflects changes in the pulse sequences and the leptin concentration levels, balancing features from both inputs.

The estimated leptin-related hidden states capture the general trend of the reconstructed plasma leptin level which displays a peak around midnight and decreases rapidly until reaching a minimum in the morning. The HSI also shows a clear distinction between wake and sleep in most results. However, some cases such as the estimated state of the pre-treatment sample of participant 16 (shown in **Figure 3**) show more oscillation than the average results. And the HSI also shows a relatively chaotic pattern. A disrupted circadian rhythm of the biological system might have been a cause of the participant’s obesity (42). Despite these cases, our comparison and test results show that the wake-sleep difference is statistically significant, and in most cases, the estimated states and the HSI have patterns similar to the results of participant 6 shown in **Figure 3**, suggesting a circadian rhythm (43). As expected, this circadian rhythm resembles that of blood leptin levels (44, 45), but it is more apparent. The near-zero HSI during most of the day in most cases reflects low basal satiety, meaning that the participant may have a good appetite under the right conditions (14). On the other hand, the high HSI during nighttime suggests high basal satiety in the participant who is unlikely to feel the need for food (14). Moreover, leptin as a satiety hormone is not only regulated by wake and sleep periods but also active and resting periods (46). The pro-satiety state also reflects this behavior. In many cases such as participant 6, both the pre-treatment and post-treatment samples indicate a high satiety after dinner and before sleep. This might suggest that the participants are relatively inactive though still awake (46). The HSI derived from the pro-satiety state reveals basal satiety status that can hardly be accurately measured through self-reporting especially in a free-living setting due to underreporting and misreporting (7).

By comparing the differences between time windows around each meal, we have found a consistent increase in the estimated state during the time windows after lunch across all participants. However, such a pattern is not apparent after dinner and does not show after breakfast. Further studies that focus on meal sizes and compositions might provide us with more details on the reason behind the discrepancy between the effects of the different meals (38). Nonetheless, circadian rhythm is dominant and can overshadow the effect of meals on long-term satiety if there is any (43). The decrease in the pro-satiety state before waking could extend to the morning and the breakfast period. The low or slowly rising HSI during these time windows further supports that food intake does not have an acute effect on the leptin-based pro-satiety state.

The original study on the dataset has found a decrease in overall mean leptin level after bromocriptine treatment (32). We expanded on this comparison by comparing the pro-satiety states estimated from pre-treatment and post-treatment data different time windows for each participant. However, the results did not show a significant change during any of the investigated time windows. Although it is shown in the previous studies (28, 32) that the mean leptin level is reduced after the treatment, bromocriptine does not affect the diurnal pattern of the pro-satiety state. Future studies including real-world monitoring studies may also implement this method in testing the effects of other medication on satiety. The proposed dynamic inference of satiety provides more details than traditional tests on related hormones or surveys.

Feeling satiated involves an integration of hormonal signals in the brain, including ghrelin secreted from the stomach and insulin from the pancreas (7, 15). Insulin has a diurnal pattern exactly in phase with that of leptin while it is more sensitive to food intake, whereas ghrelin has a pattern reciprocal to that of insulin (7, 47). This study focused on leptin and its link to the long-term aspect of the satiety cascade (13). Based on our satiety inference scheme, a multimodal estimator can be developed to incorporate more related biosignals such as insulin, and ghrelin, which have short reaction times to food intake (14). Nonetheless, using an unimodal approach, we isolate the hormone and may have a clearer understanding of its specific effect on human homeostasis. To fully capture the satiety felt by a person, downstream neural links need to be studied. In other words, how leptin and other hormones signaling in the hypothalamus leads to behaviors such as food seeking, food consumption, and meal termination requires further investigation (48). Both a unimodal analysis of each involved biosignals and a multimodal analysis of the biosignals combined might be needed for determining their exact roles in appetite control.

The presented unimodal algorithm may have better time efficiency when implemented in real-world applications like health monitoring devices. The leptin data collection required measuring plasma leptin concentrations from blood samples every 10 minutes for 24 hours (32). The details are presented in the Methods section. This invasive way of data collection is not practical for long-term monitoring and the development of an automated wearable device. To increase temporal resolution, a higher frequency of sample collection might be preferred. Thus, non-invasive measurements are essential for a potential wearable device. Recently, studies have applied new technologies to measure and monitor glucose, glucocorticoids, and other biosignals in non-invasive or minimally invasive ways, such as electrochemical continuous glucose monitoring (CGM) (49) and sweat-sensor based CGM (50). Other CGM schemes include using optics or microwaves (51). In addition, U-RHYTHM, a portable device that collects interstitial fluid is capable of measuring glucocorticoids and other hormones for a long period (52, 53). These examples have shown that a better leptin measuring scheme is possible, and implementing our method in a wearable device is achievable. Since our long-term satiety estimator assumed relatively simple links between observations and the latent state, the estimation process is fast and can be done in real time. However, the deconvolution for recovering the underlying pulses may take a longer time, but a near real-time deconvolution algorithm is also possible (54). In addition, the presented state estimation algorithm is flexible and can be customized depending on the individual by setting different hyperparameters (details on hyperparameters are available in the **Supplementary Materials**). This could be a crucial function for wearable devices that can adapt to users with different health conditions.

In conclusion, this study applied an estimator to infer the 24-hour basal satiety using leptin secretory pulses and the continuous leptin levels. The estimated hidden states display a circadian rhythm with a prominent increase during nighttime. This hidden state is an indicator of long-term basal satiety grounded in the assumed causal link between the hidden state and leptin secretion as well as between leptin secretion and the pro-satiety state (7, 13). Despite limited sample size, our method presents the feasibility of applying a Bayesian framework to track satiety, demonstrating its practicality in revealing person-specific hidden health status and providing a new direction in drug testing and patient monitoring for future studies with larger cohort sizes. Future studies may also incorporate other satiety-related hormones such as ghrelin and insulin and apply both a unimodal and a multimodal framework to reflect a more complete spectrum of satiety. Furthermore, the same method can be applied to investigate the differences in the pro-satiety state and HSI across different weight groups. The results might help design a more personalized treatment of satiety-related diseases and diets for general health improvement. larger groups of participants with different body weights should be studied under the same framework to consider its correspondence with body weight. Last but not least, with the rapid development of minimally invasive and portable devices, the proposed satiety inference method contributes to a real-world implementation of automated monitoring and medication testing for satiety and potentially other latent states.

## Data Availability

The original contributions presented in the study are included in the article/**Supplementary Material**. Further inquiries can be directed to the corresponding author.
