# Supplementary material for: A leptin-based Bayesian inference of a pro-satiety state reflects a basal circadian rhythm in women with obesity
Source: Front Endocrinol (Lausanne). 2025 Sep 16;16:1638568. doi: 10.3389/fendo.2025.1638568 (PMC12479250; doi:10.3389/fendo.2025.1638568)
Supplement: Supplementary file 1 [file DataSheet1.pdf]

Supplementary Material

1 HYPERPARAMETER TUNING

Ideally, since fitting to one input would increase the estimated variance for the other input, the algorithm can automatically prevent overfitting by minimizing the cost function that takes both variances into account. However, the presented estimator tends to overfit to s_j because decreasing σ_ω^2 does not increase σ_ε^2 as much, leading to a very small σ_ω^2 as the algorithm converges. To address this issue, we applied a similar method employed in the previously mentioned state estimation study Wickramasuriya et al. (2023). The method involves tuning three hyperparameters: λ, $\sigma^2_{\omega,init}$ and $\sigma^2_{\omega,stop}$. λ is a gain applied to each step of the update to control the rate at which the model fits to s_j. $\sigma^2_{\omega,init}$ and $\sigma^2_{\omega,stop}$ are the initialization and stopping criterion for σ_ω^2. Although some general pattern is expected, the MPP and plasma leptin level profiles vary from case to case. To address the inter-participant differences, we set $\sigma^2_{\omega,init}$ to the variance of continuous observation s_j and $\sigma^2_{\omega,stop}$ to a fraction α times the variance of s_j. When σ_ω^2 becomes smaller than $\sigma^2_{\omega,stop}$, the algorithm stops to prevent overfitting. α is set to 0.6 in the shown results. With different α selections, the estimation result can potentially reflect different features. The step size λ also leaves freedom for the estimator to reveal different details. λ is chosen to be 0.5 in the results shown.

For overall data analysis and comparison in this study, we applied the same hyperparameters for all participants. For future studies, however, tuning these hyperparameters for an emphasis on different features may provide more information on the person-specific health condition and help pinpoint causes of potential abnormalities. This will be especially useful in future multimodal designs where tuning the hyperparameters can effectively isolate influences from different biosignals or show a balanced mixture of them for an overall impression.

2 SUPPLEMENTARY TABLES AND FIGURES

2.1 Figures

Figures of results from participants 1-18 are presented, and they share the same caption as follows:

(a) Plasma leptin measurements (black) and reconstructed plasma leptin concentration level (red). (b) Leptin secretory pulse events. (c) Estimated satiety state with a 95% confidence interval. (d) Probability of leptin pulse occurrence with a 95% confidence interval. (e) The High Satiety Index (HSI). Meal periods are highlighted in red, and sleep period is highlighted in green. Est: estimation. Cont: continuous observation. MPP: marked point process.

REFERENCES

Wickramasuriya DS, Khazaei S, Kiani R, Faghih RT. A bayesian filtering approach for tracking sympathetic arousal and cortisol-related energy from marked point process and continuous-valued observations. *IEEE Access* **11** (2023) 137204–137247.

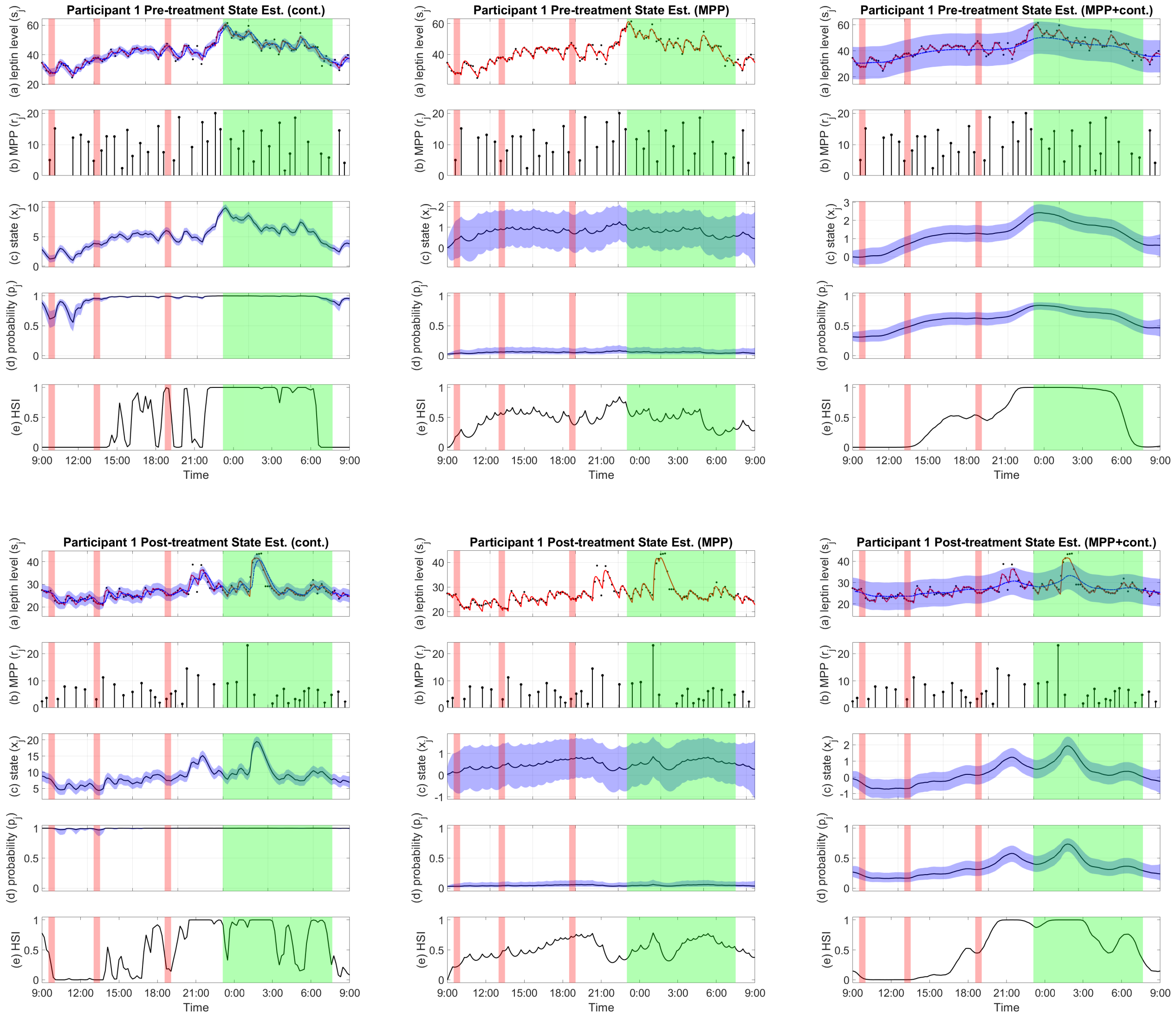

Figure S1. Participant 1 pre-treatment and post-treatment pro-satiety state estimation results using different estimators.

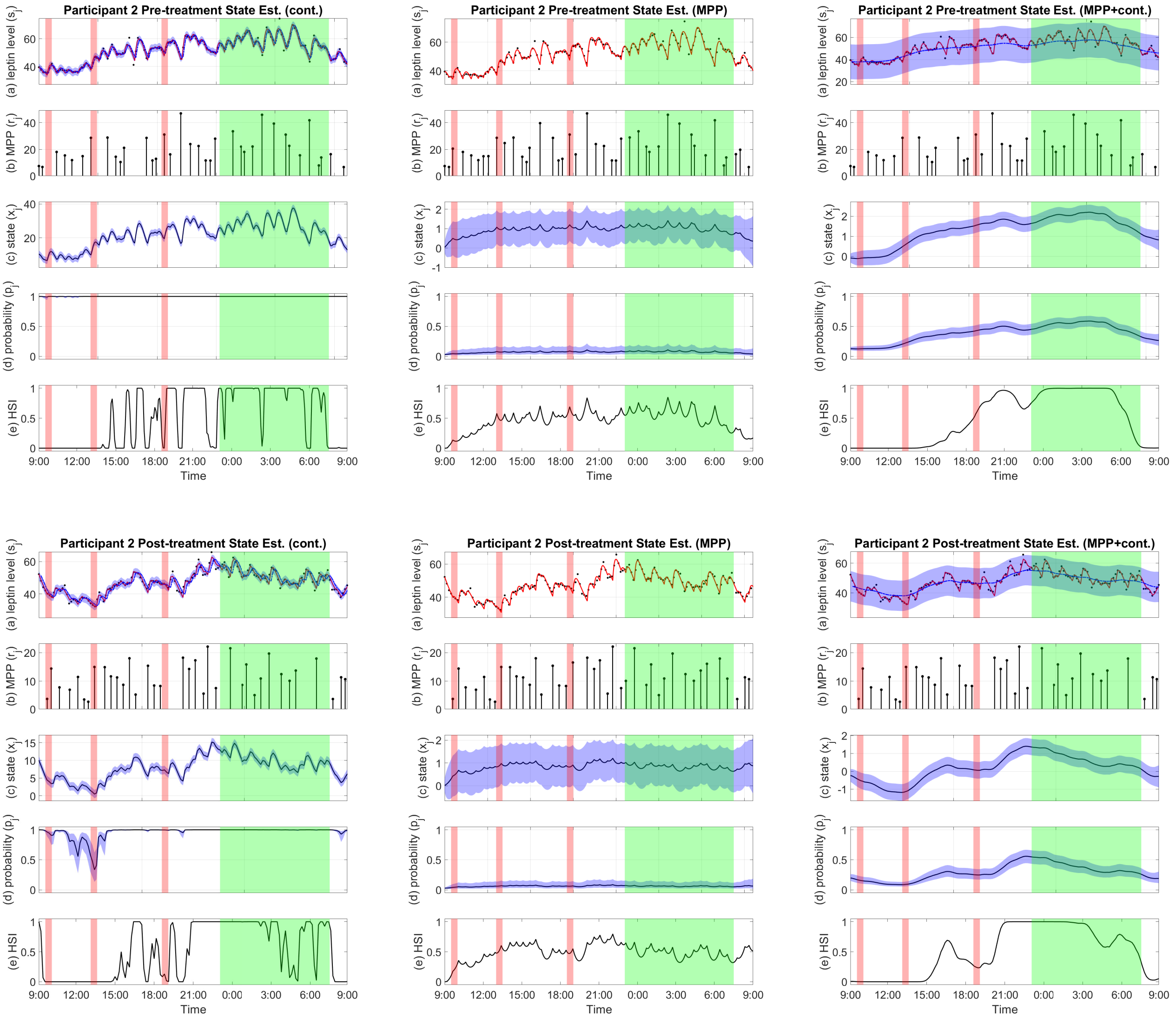

Figure S2. Participant 2 pre-treatment and post-treatment pro-satiety state estimation results using different estimators.

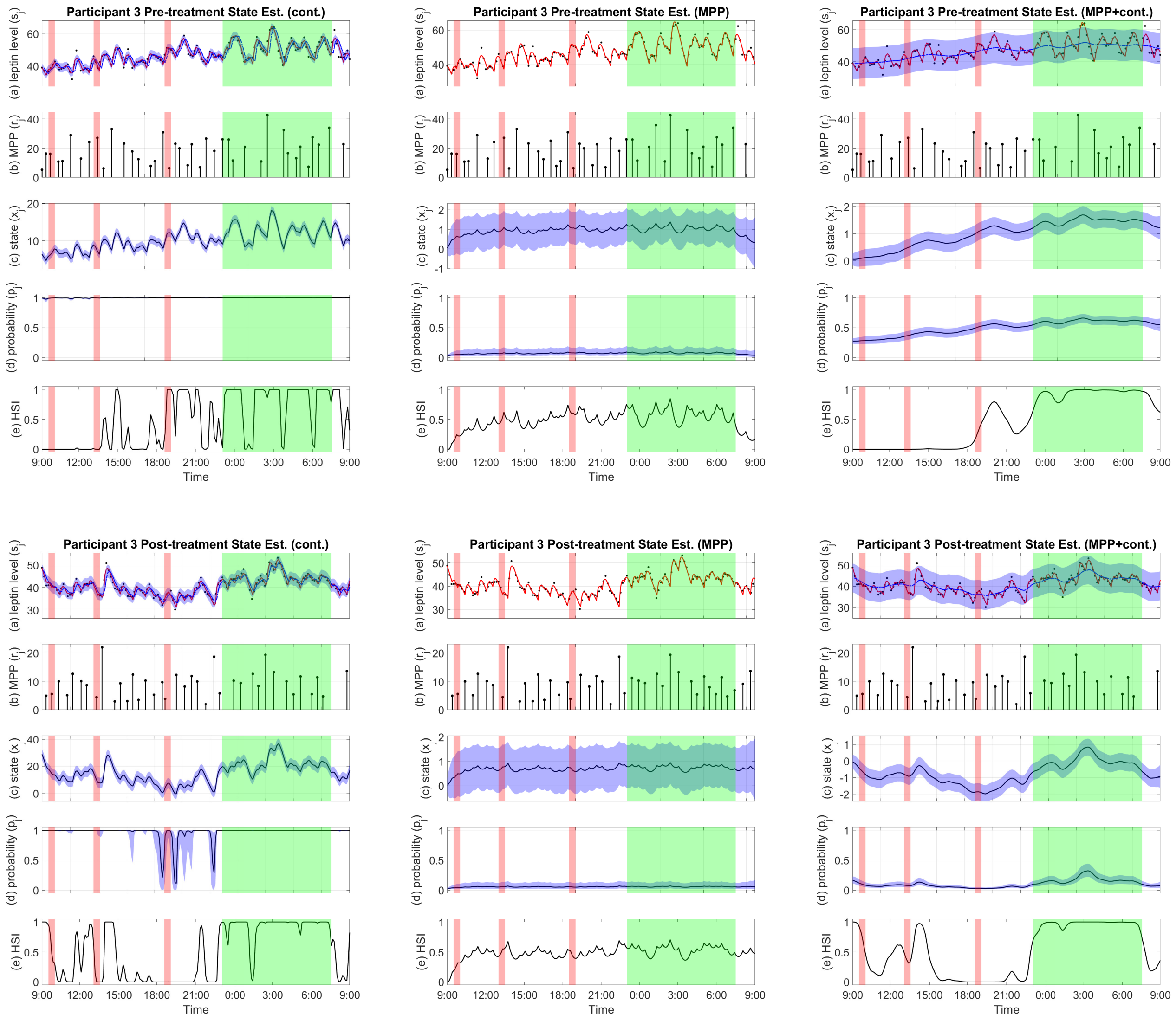

Figure S3. Participant 3 pre-treatment and post-treatment pro-satiety state estimation results using different estimators.

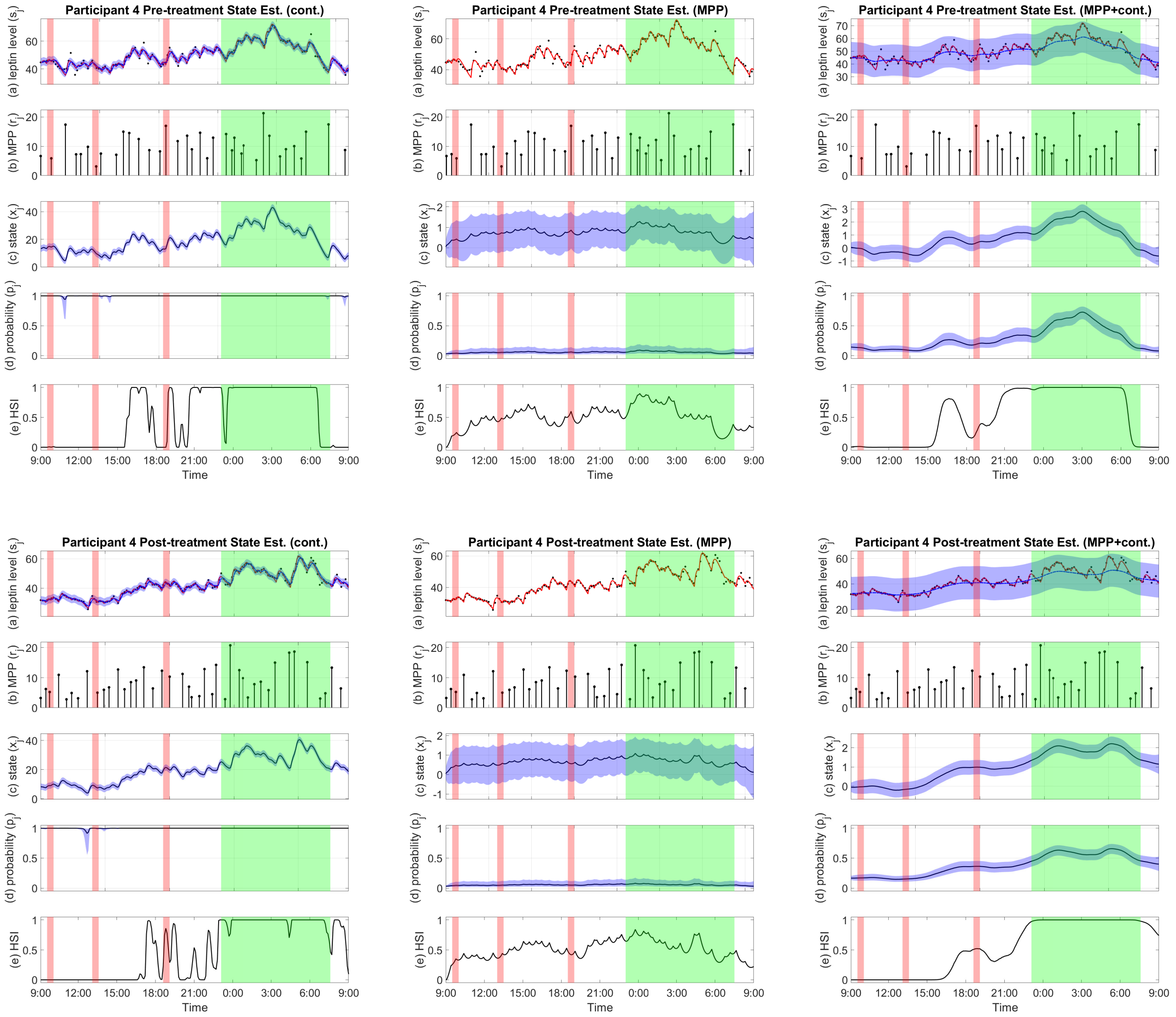

Figure S4. Participant 4 pre-treatment and post-treatment pro-satiety state estimation results using different estimators.

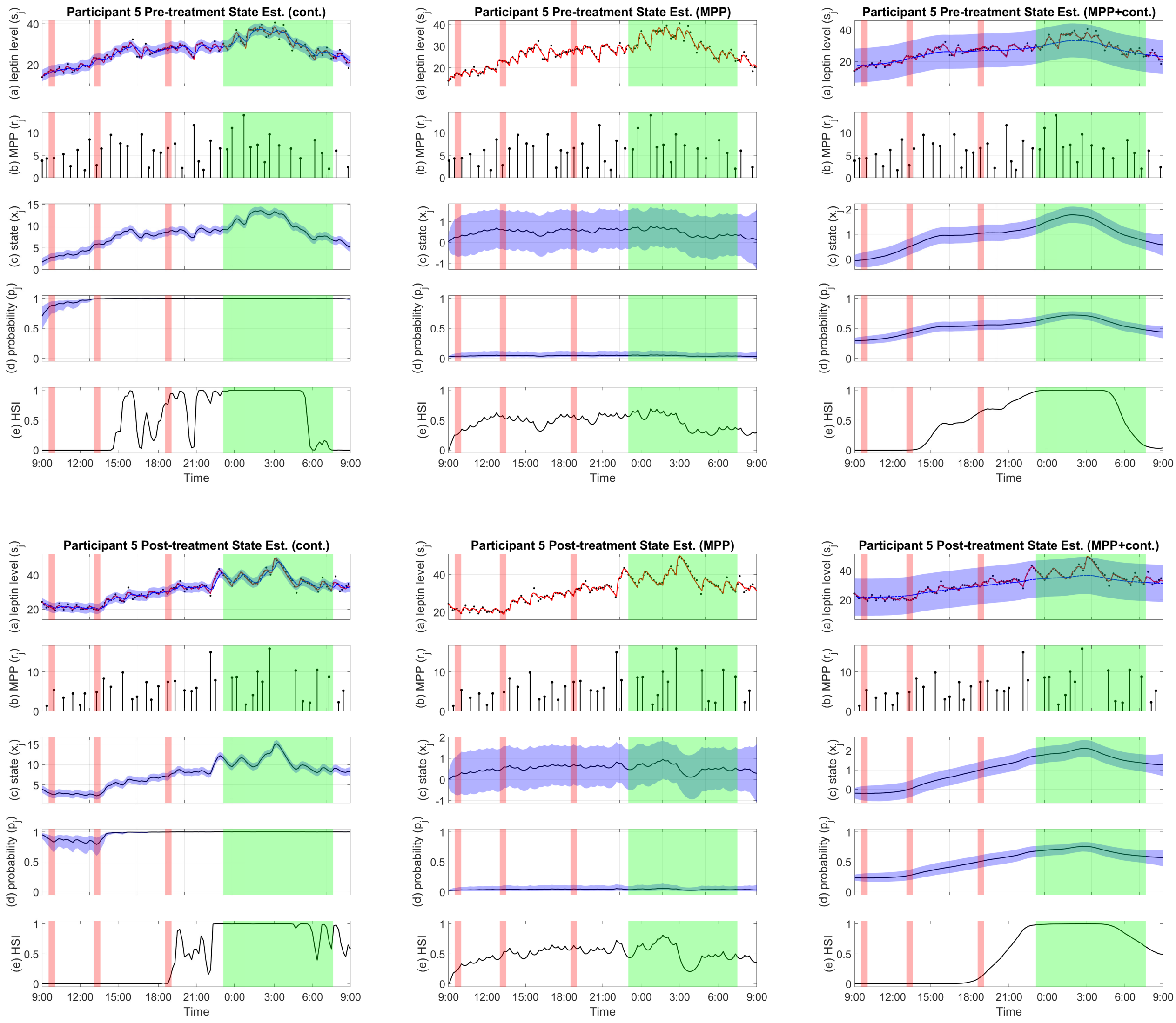

Figure S5. Participant 5 pre-treatment and post-treatment pro-satiety state estimation results using different estimators.

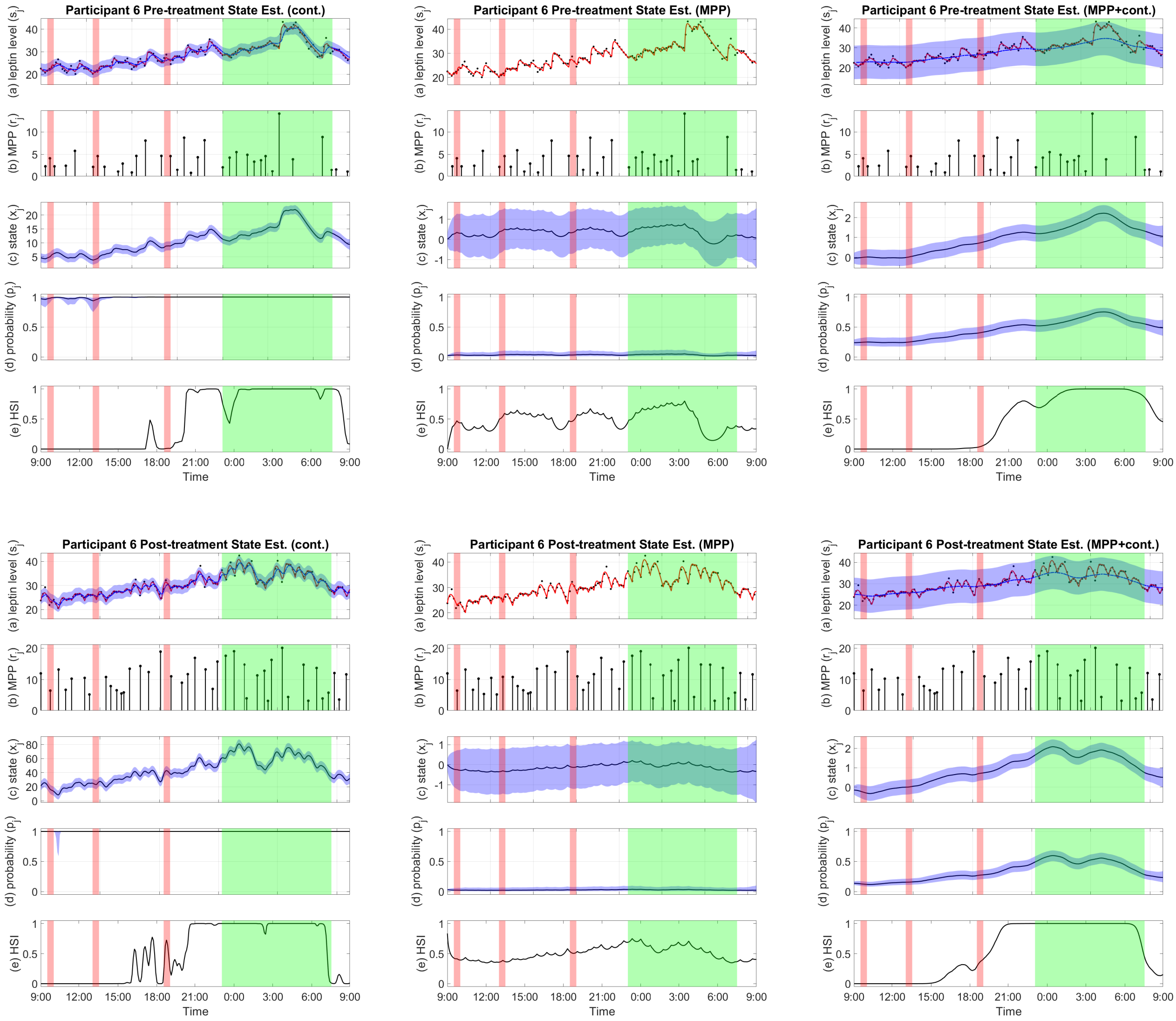

Figure S6. Participant 6 pre-treatment and post-treatment pro-satiety state estimation results using different estimators.

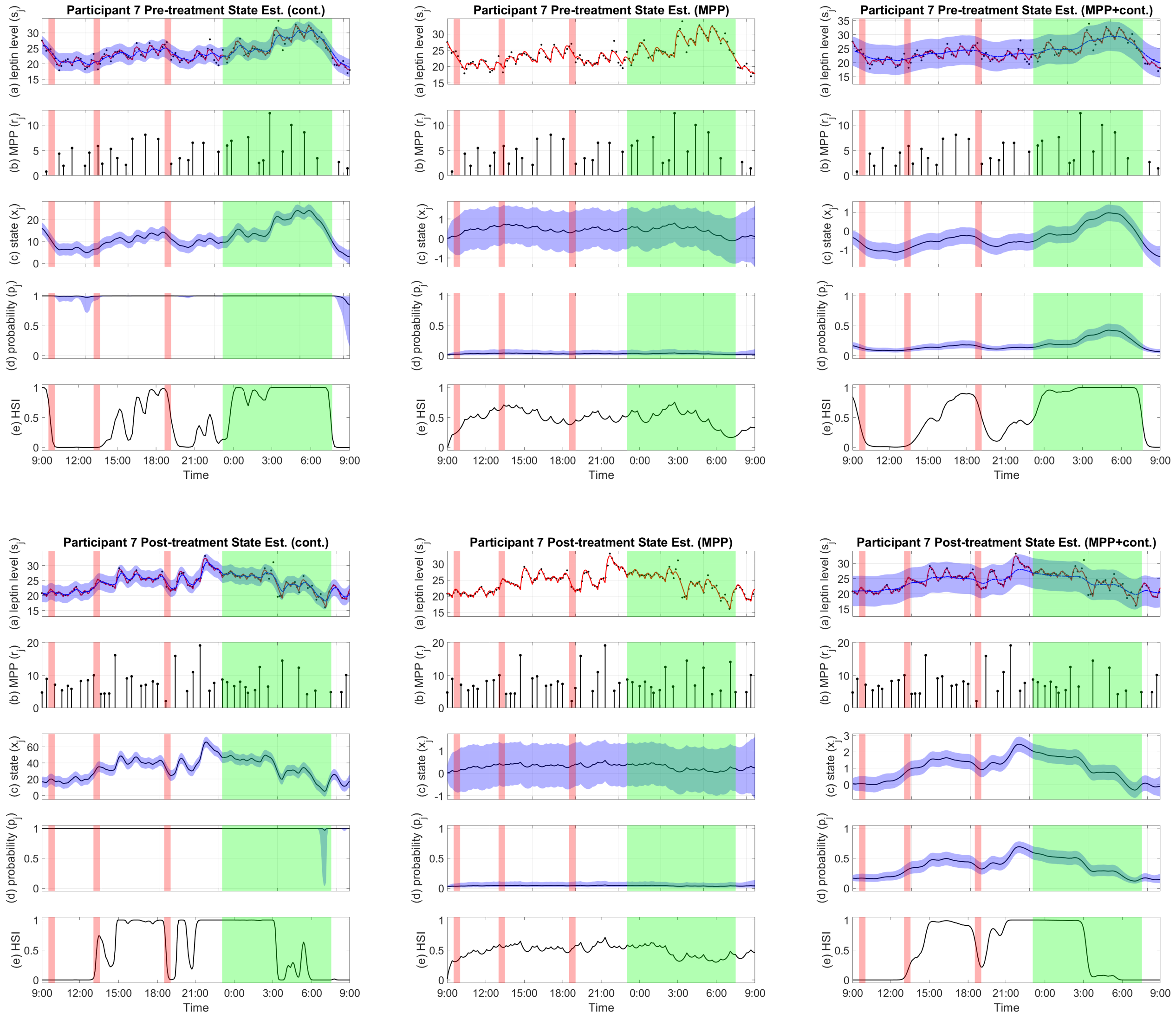

Figure S7. Participant 7 pre-treatment and post-treatment pro-satiety state estimation results using different estimators.

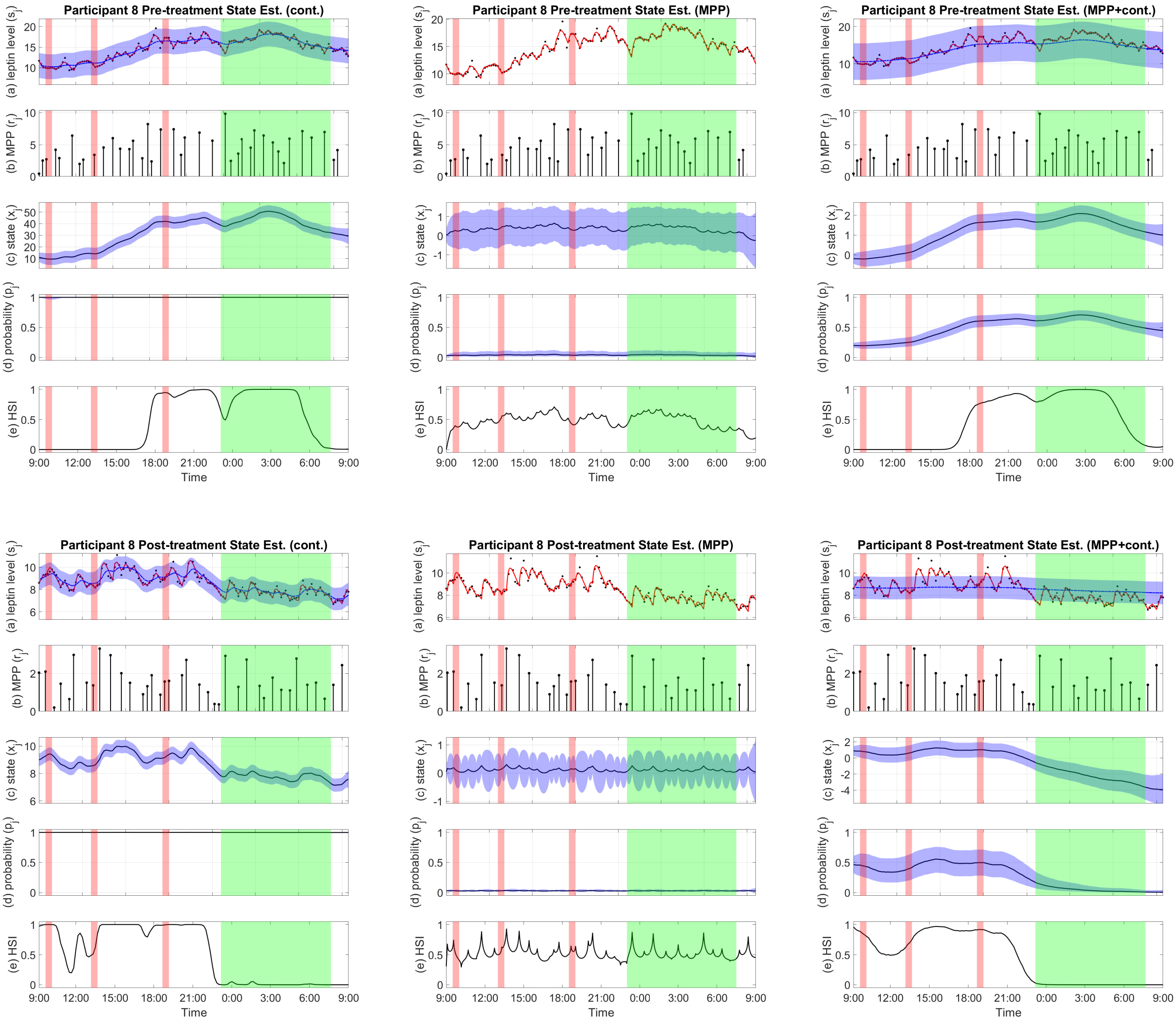

Figure S8. Participant 8 pre-treatment and post-treatment pro-satiety state estimation results using different estimators.

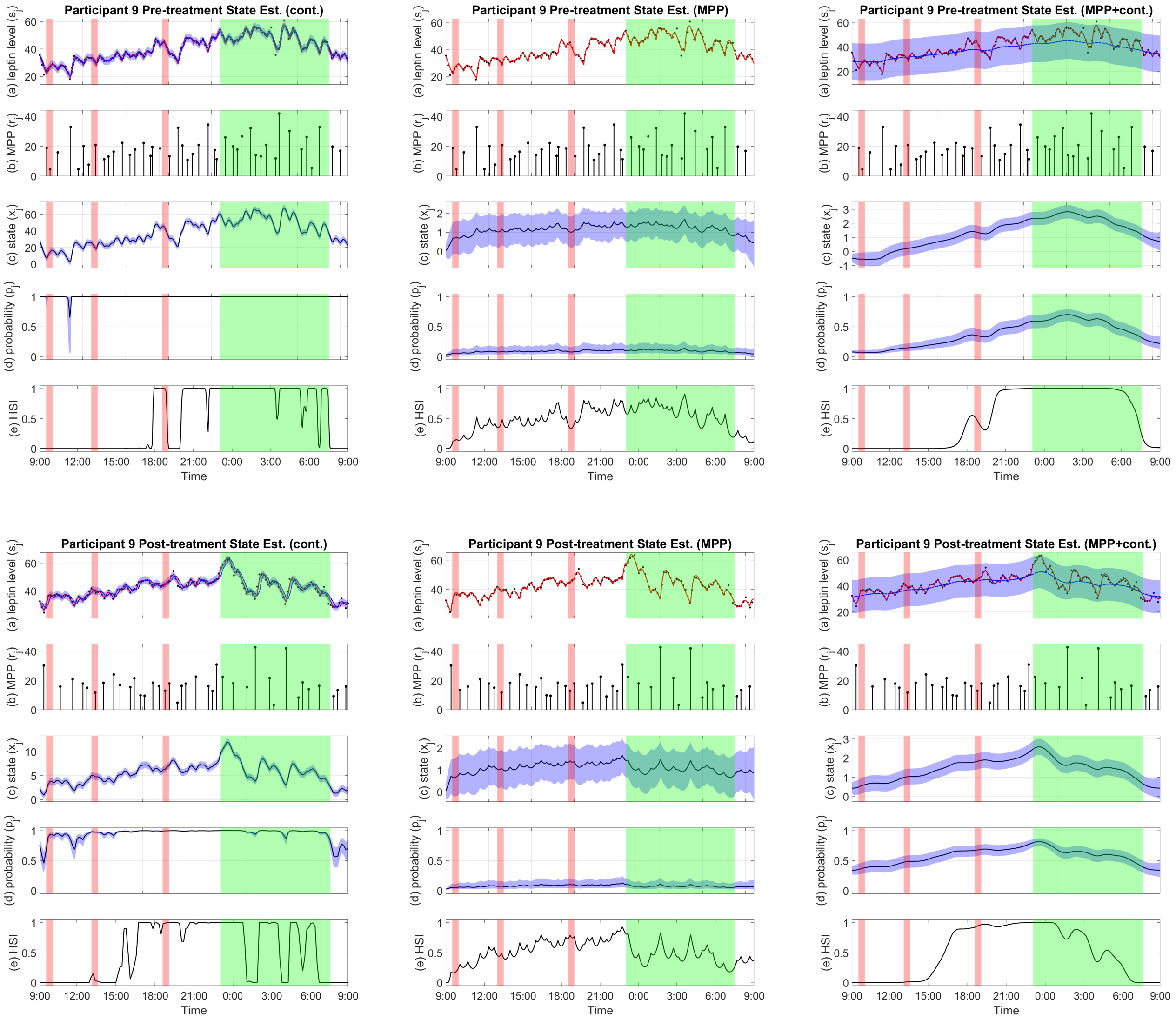

Figure S9. Participant 9 pre-treatment and post-treatment pro-satiety state estimation results using different estimators.

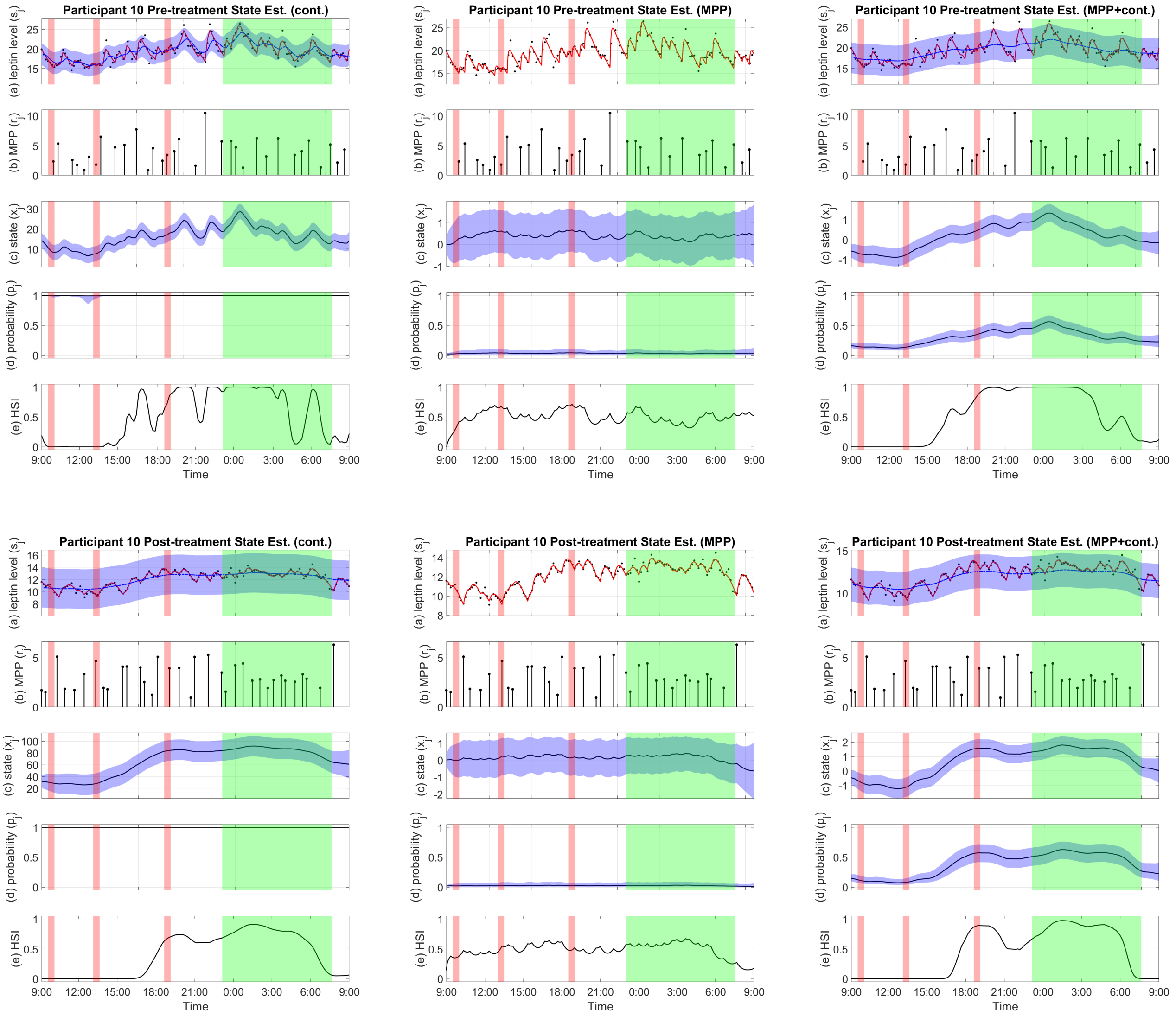

Figure S10. Participant 10 pre-treatment and post-treatment pro-satiety state estimation results using different estimators.

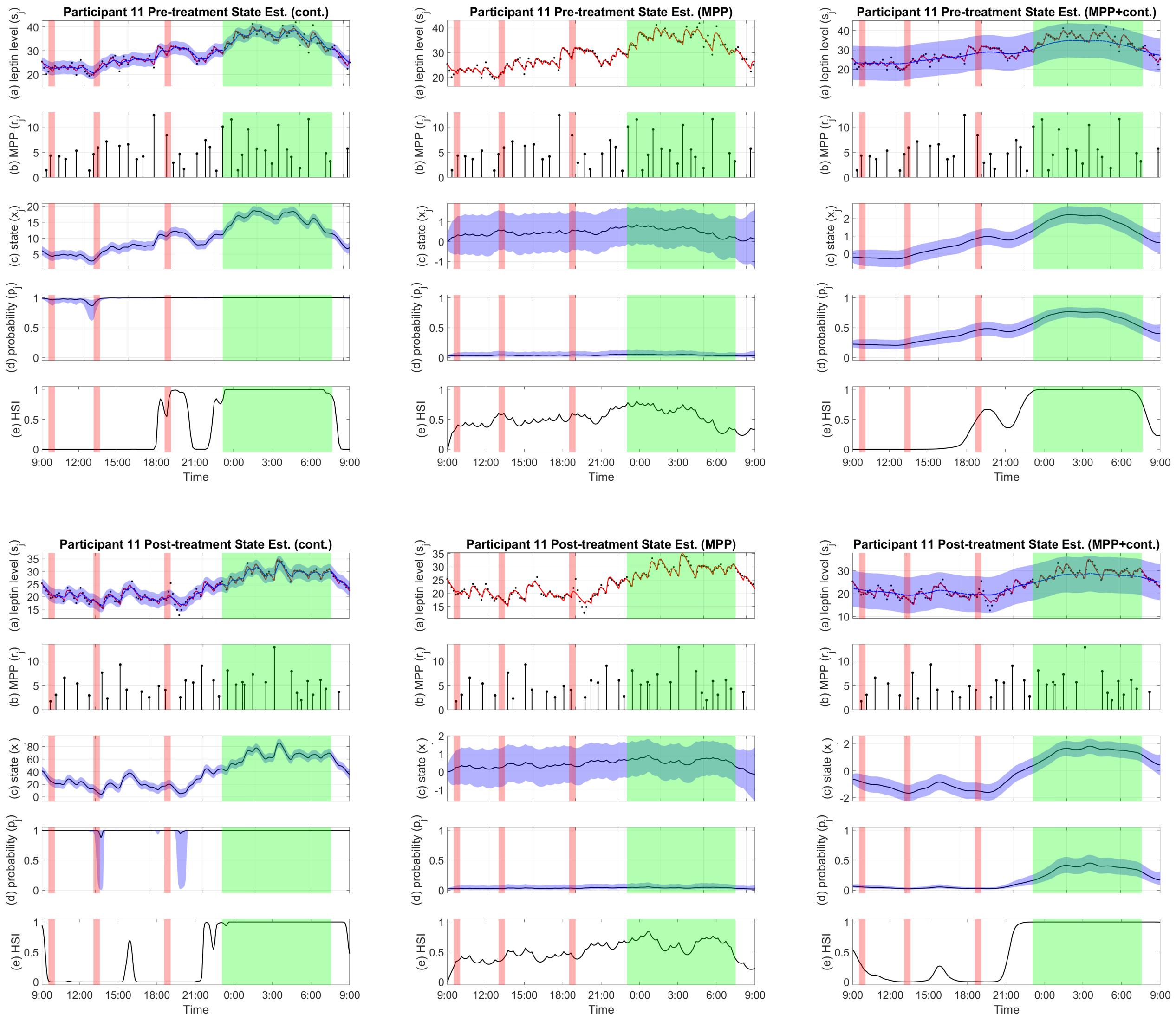

Figure S11. Participant 11 pre-treatment and post-treatment pro-satiety state estimation results using different estimators.

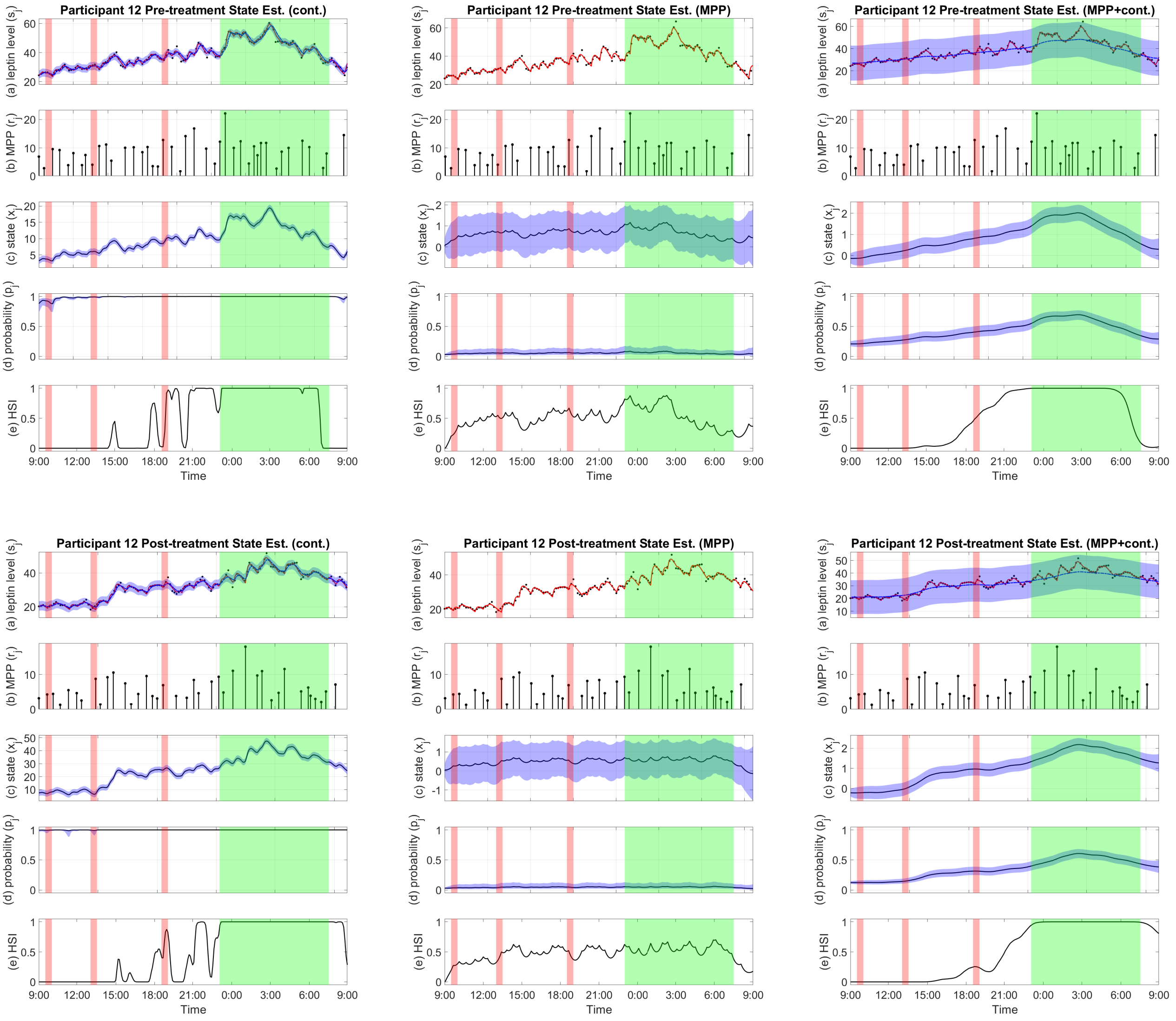

Figure S12. Participant 12 pre-treatment and post-treatment pro-satiety state estimation results using different estimators.

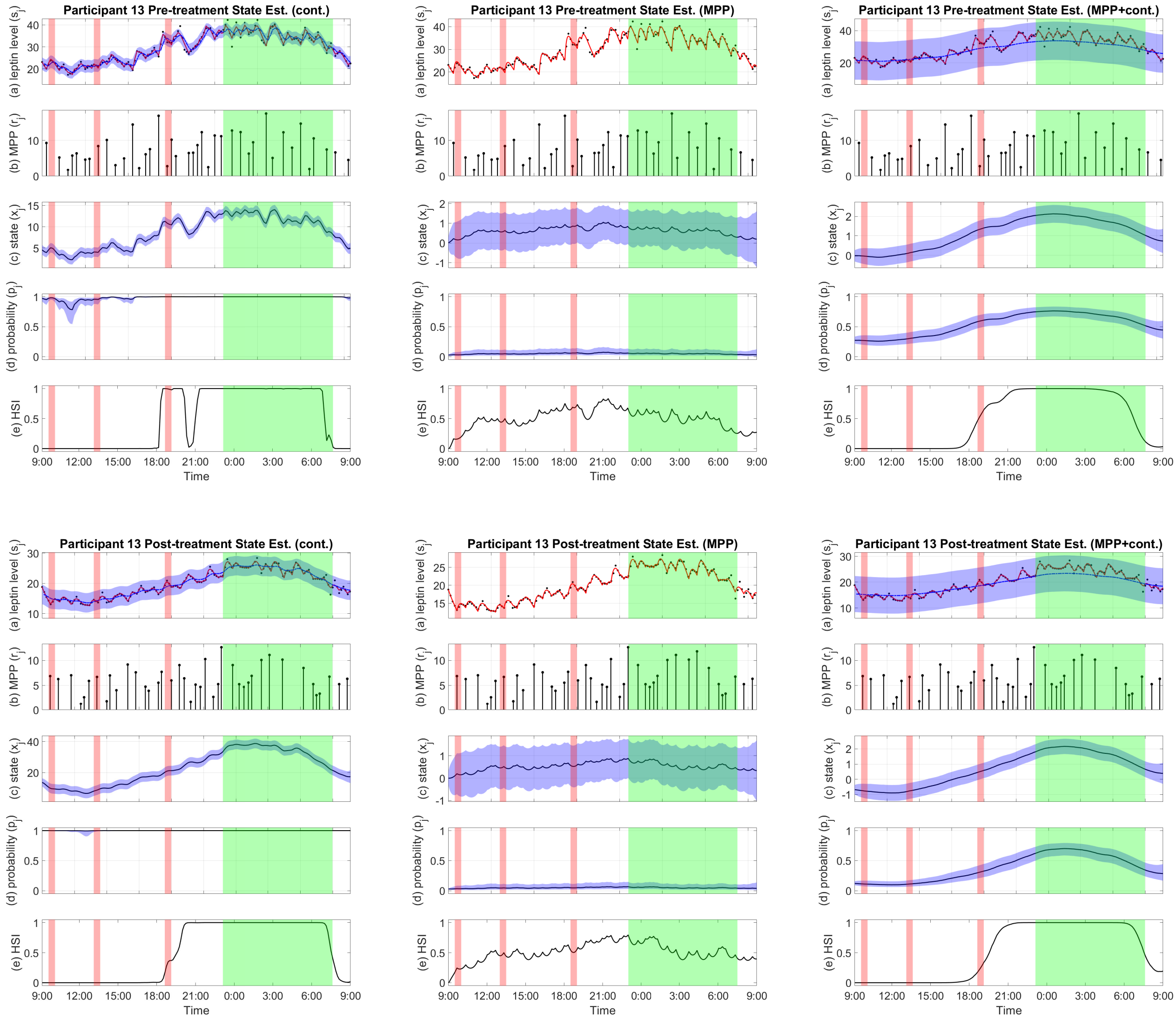

Figure S13. Participant 13 pre-treatment and post-treatment pro-satiety state estimation results using different estimators.

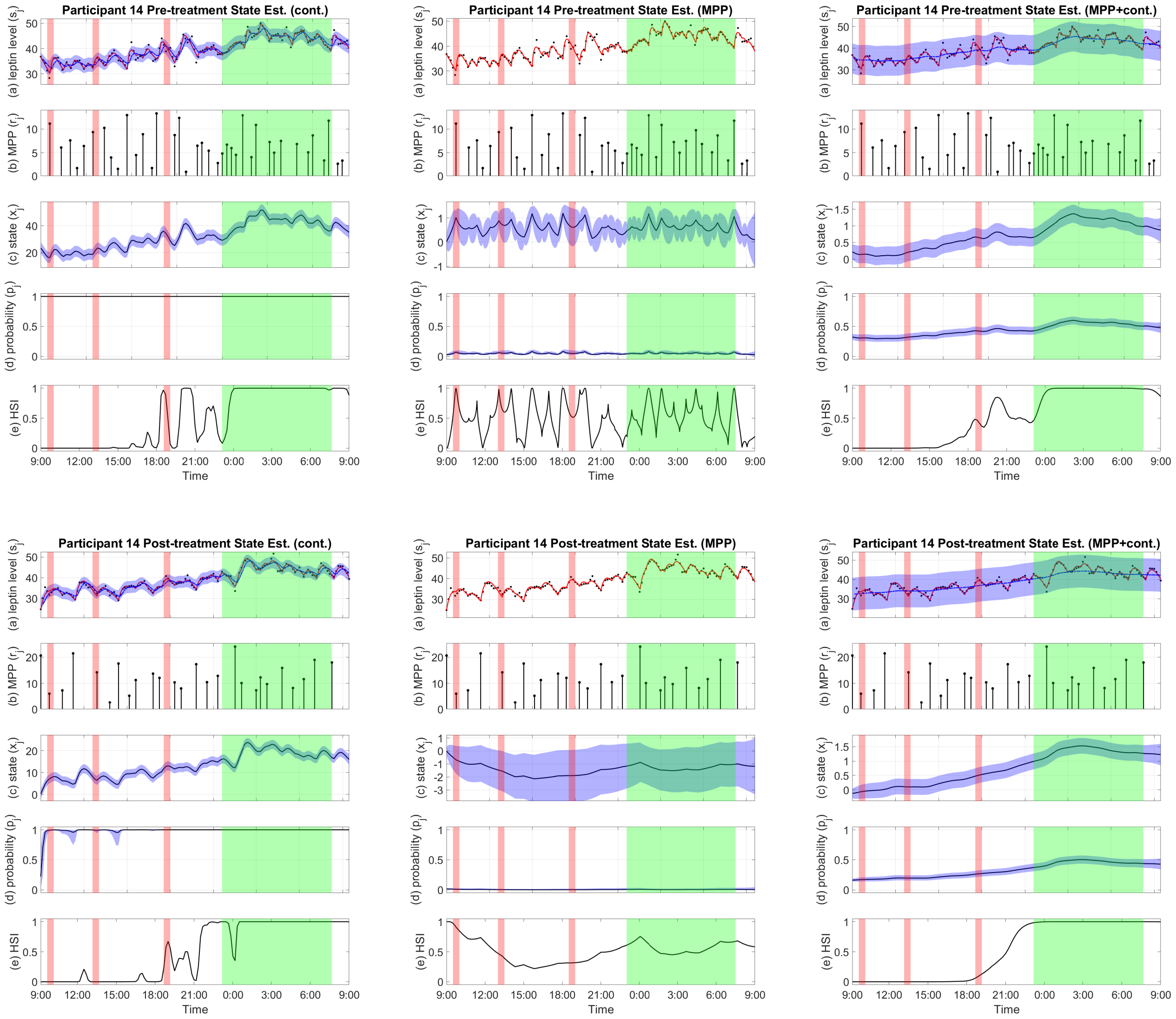

Figure S14. Participant 14 pre-treatment and post-treatment pro-satiety state estimation results using different estimators.

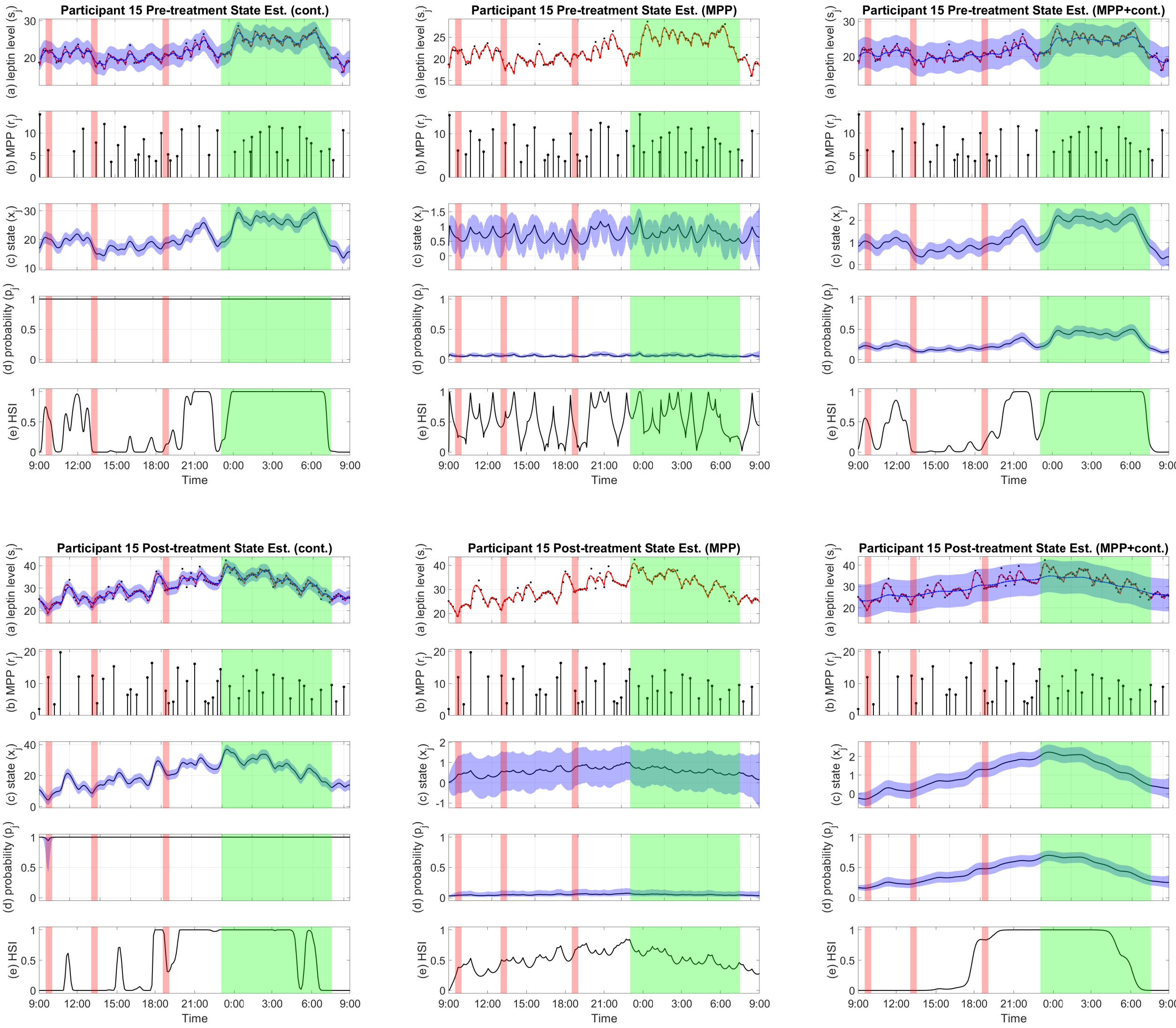

Figure S15. Participant 15 pre-treatment and post-treatment pro-satiety state estimation results using different estimators.

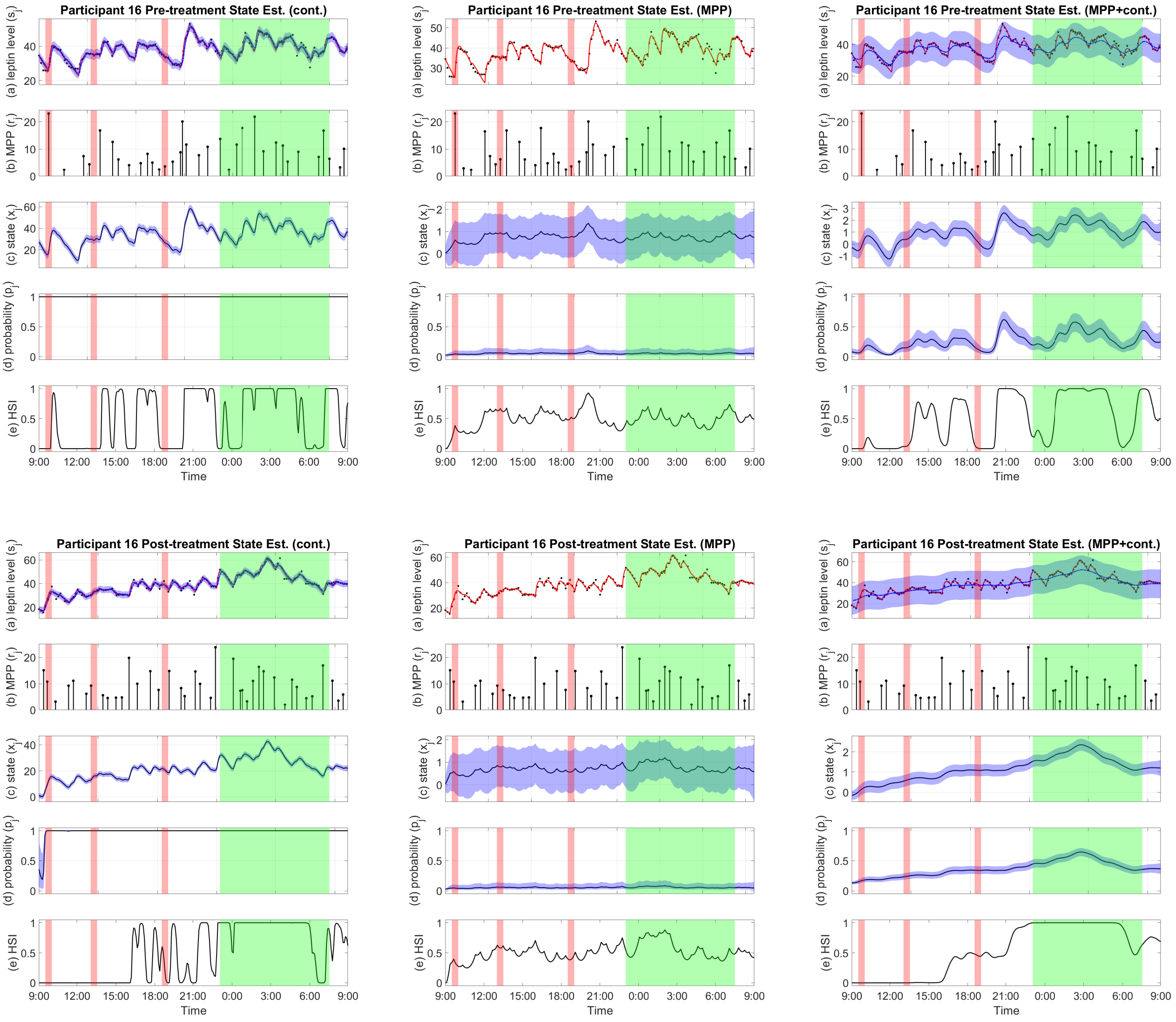

Figure S16. Participant 16 pre-treatment and post-treatment pro-satiety state estimation results using different estimators.

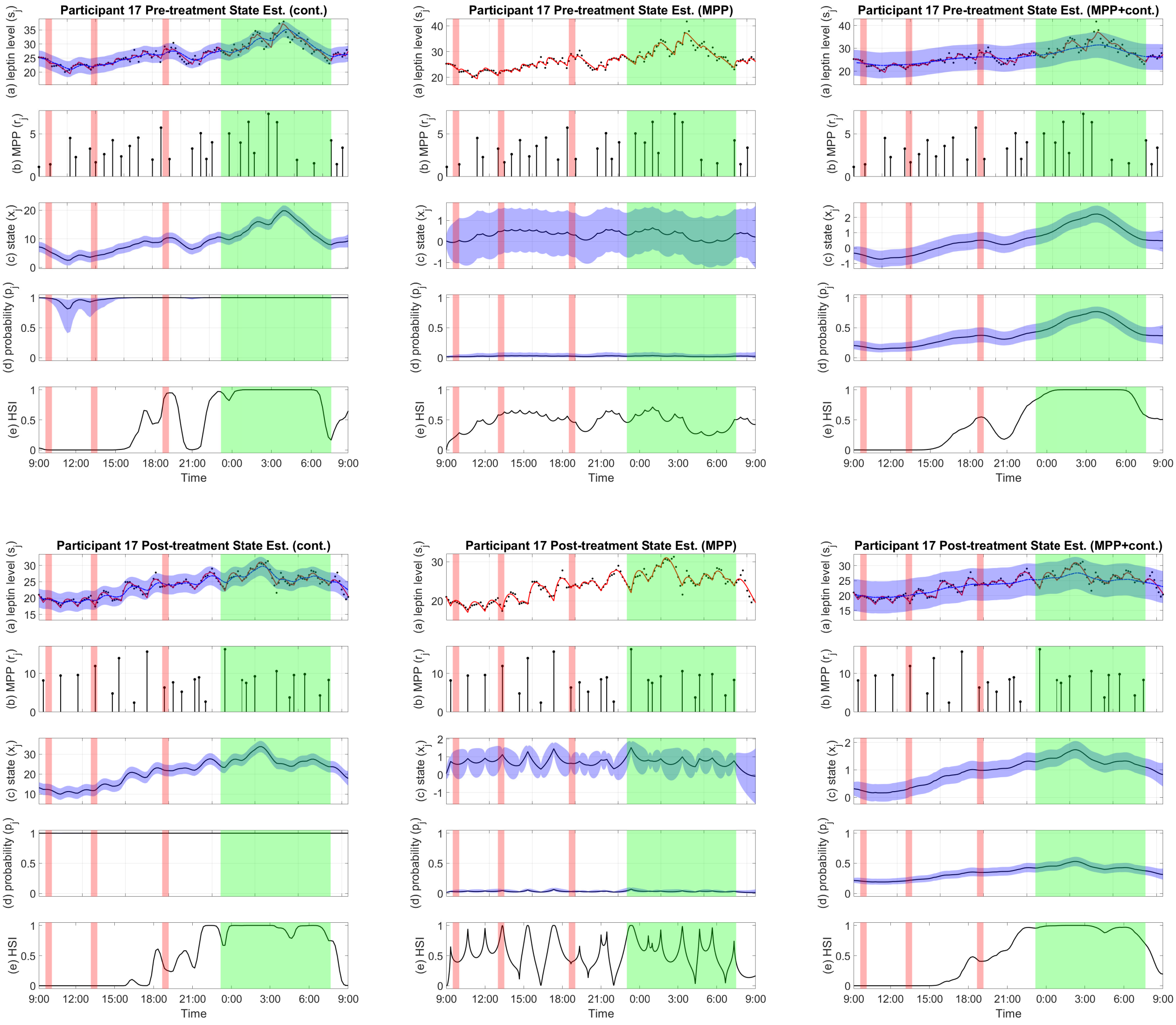

Figure S17. Participant 17 pre-treatment and post-treatment pro-satiety state estimation results using different estimators.

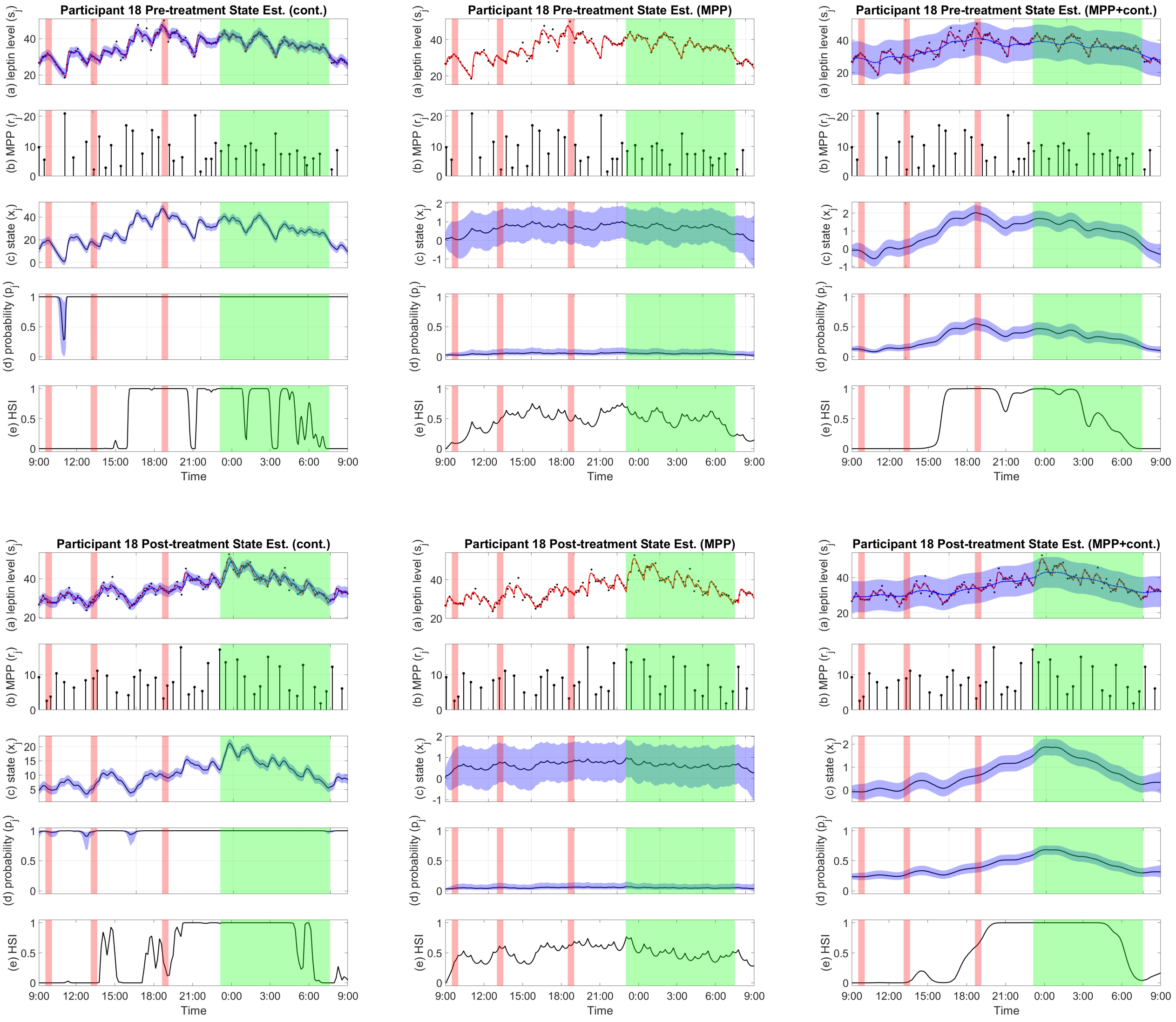

Figure S18. Participant 18 pre-treatment and post-treatment pro-satiety state estimation results using different estimators.
